# Atención integral a la salud de adolescentes y jóvenes desde la perspectiva de profesionales de atención primaria, San Pablo, Brasil

**DOI:** 10.18294/sc.2025.5419

**Published:** 2025-02-21

**Authors:** José Ricardo de Carvalho Mesquita Ayres, Valéria Monteiro Mendes, Isabella Silva de Almeida, Macarena Urrestarazu Devincenzi, Jamile Silva Guimarães, Gabriela Junqueira Calazans, Pamela Cristina Bianchi, Alberto Shodi Yamashiro, Grupo de Pesquisa em Saúde de Jovens e Direitos Humanos (SJ&DH) 9

**Affiliations:** 1 Libre-Docencia. Profesor titular, Departamento de Medicina Preventiva, Faculdade de Medicina, Universidade de São Paulo, São Paulo, Brasil. jrcayres@usp.br Universidade de São Paulo Departamento de Medicina Preventiva Faculdade de Medicina Universidade de São Paulo São Paulo Brazil jrcayres@usp.br; 2 Doctora en Salud Pública. Posdoctoranda en Salud Colectiva, Departamento de Medicina Preventiva, Faculdade de Medicina, Universidade de São Paulo, São Paulo, Brasil. isa.almeida@alumni.usp.br Universidade de São Paulo Departamento de Medicina Preventiva Faculdade de Medicina Universidade de São Paulo São Paulo Brazil isa.almeida@alumni.usp.br; 3 Doctora en Ciencias de la Salud. Posdoctoranda en Salud Colectiva, Departamento de Medicina Preventiva, Faculdade de Medicina, Universidade de São Paulo, São Paulo, Brasil. diego.salusso@huesped.org.ar Universidade de São Paulo Departamento de Medicina Preventiva Faculdade de Medicina Universidade de São Paulo São Paulo Brazil diego.salusso@huesped.org.ar; 4 Posdoctorada en salud Colectiva. Profesora asociada, Instituto de Saúde e Sociedade, Universidade Federal de São Paulo, São Paulo, Brasil. macarena.devincenzi@unifesp.br Universidade Federal de São Paulo Instituto de Saúde e Sociedade Universidade Federal de São Paulo São Paulo Brazil macarena.devincenzi@unifesp.br; 5 Doctora en Salud Pública. Profesora adjunta, Faculdade de Medicina, Universidade Federal de Minas Gerais, Belo Horizonte, Brasil. mile.guimaraes@gmail.com Universidade Federal de Minas Gerais Faculdade de Medicina Universidade Federal de Minas Gerais Belo Horizonte Brazil mile.guimaraes@gmail.com; 6 Doctora en Medicina Preventiva. Profesora, Instituto de Psicologia, Universidade de São Paulo, São Paulo, Brasil. gajuca@usp.br Universidade de São Paulo Instituto de Psicologia Universidade de São Paulo São Paulo Brazil gajuca@usp.br; 7 Doctora en Terapia Ocupacional. Profesora adjunta, Instituto de Saúde e Sociedade, Universidade Federal de São Paulo, São Paulo, Brasil. pamela.bianchi@unifesp.br Universidade Federal de São Paulo Instituto de Saúde e Sociedade Universidade Federal de São Paulo São Paulo Brazil pamela.bianchi@unifesp.br; 8 Magíster en Tecnologías y Atención a la Salud. Doctorando en Educación, Universidade Federal de São Carlos, Sorocaba, Brasil. alberto190@gmail.com Universidade Federal de São Carlos Universidade Federal de São Carlos Sorocaba Brazil alberto190@gmail.com; 9 Grupo interdisciplinario e interinstitucional (USP, UNIFESP, UFSCAR, UFMG, UFR/Mato Grosso), con sede en el Departamento de Psicología Social, Universidad de São Paulo, Brasil. tematico2017pst@usp.br Universidad de São Paulo Departamento de Psicología Social Universidad de São Paulo Brazil tematico2017pst@usp.br

**Keywords:** Adolescentes, Adulto Joven, Integralidad en Salud, Atención Primaria de Salud, Derechos Humanos, Brasil

## Abstract

La integralidad es un principio recomendado a nivel global para una atención a la salud eficaz, especialmente en el caso de adolescentes y jóvenes, cuyas necesidades asocian aspectos biomédicos con procesos socioculturales de construcción de subjetividad y ejercicio de la ciudadanía. Sin embargo, existen importantes desafíos para su implementación efectiva. Este estudio tiene como objetivo identificar aquellos aspectos del trabajo realizado en unidades básicas de salud del estado de São Paulo, Brasil, que los profesionales consideran como facilitadores u obstáculos para la producción de un cuidado integral a la salud de adolescentes y jóvenes. Se trata de un estudio cualitativo llevado a cabo entre enero y noviembre de 2023, en el que se realizó observación del cotidiano de trabajo y entrevistas semiestructuradas a 73 profesionales de la salud. Se destaca el predominio de acciones descontextualizadas, fragmentadas y prescriptivas, en un contexto de carencias de recursos y de articulaciones intersectoriales. Se sostiene la necesidad de que adolescentes y jóvenes participen como sujetos de derechos en la construcción de acciones y políticas intersectoriales de salud.

## INTRODUCCIÓN

La integración de servicios y niveles de atención, centrada en las necesidades de salud de individuos y poblaciones en sus diversos y singulares contextos sanitarios y socioculturales, es recomendada globalmente como un elemento fundamental para la construcción de sistemas de salud más eficaces. Entendida como una atención a la salud organizada de manera que las personas reciban un continuo de promoción de la salud, prevención de enfermedades, diagnóstico, tratamiento, manejo de enfermedades, rehabilitación y cuidados paliativos, coordinados entre los diferentes niveles y espacios de atención dentro y fuera del sector de la salud, y de acuerdo con sus necesidades a lo largo de la vida, existen evidencias de que la integralidad favorece mejoras en los resultados clínicos y de salud, aumenta la alfabetización en salud y el autocuidado, genera mayor satisfacción en las personas usuarias y profesionales, incrementa la eficiencia de los servicios y reduce los costos globales^1^.

Diversos países de América Latina han adoptado la perspectiva de la integralidad como un eje central para la organización de la atención a la salud, en sus diversas dimensiones de gestión del cuidado, formulación de políticas, promoción y protección de derechos, y formación de recursos humanos, particularmente en lo que respecta a la salud de adolescentes y jóvenes^2^^,^^3^^,^^4^^,^^5^^,^^6^.

En Brasil, la integralidad se ha constituido como uno de los principios doctrinarios del Sistema Único de Salud (SUS). De manera articulada con los principios de universalidad -es decir, garantizando el acceso a todas las personas que viven en el país- y equidad -priorizando recursos según criterios de necesidad-, el principio de integralidad resulta fundamental para el cumplimiento de los propósitos de promoción, protección y recuperación de la salud de la población brasileña^7^. Si bien se trata de un término impreciso y polisémico, existe consenso en que la integralidad expresa el compromiso del SUS con una atención a la salud que no se limita a la provisión de asistencia en situaciones de enfermedad, sino que se interesa activamente en la producción de salud, en el sentido amplio del buen vivir de las personas y comunidades en sus diversos contextos socioculturales y conforme a sus legítimos valores, intereses y, especialmente, sus derechos^8^.

Si la integralidad es necesaria para la atención a la salud de la población en su conjunto, resulta aún más fundamental para adolescentes y jóvenes, es decir, personas de entre 10 y 24 años. Esto se debe a que, debido a las características físicas y psicosociales de este segmento poblacional, cuestiones interrelacionadas y complejas, como las transformaciones corporales, la construcción de identidad y autonomía, el ejercicio de la sexualidad, los cambios en los perfiles de sociabilidad, entre otras, requieren una mirada amplia y sensible para atender un conjunto específico de demandas en las políticas y acciones de salud^9^.

La atención primaria, al ser la vía privilegiada de entrada al SUS, al ser accesible y resolutiva para cerca del 85% de las demandas de atención, y también al ser el espacio tecnológicamente más adecuado para la implementación de una atención integral a la salud, desempeña un papel de gran relevancia en la atención integral a la salud de adolescentes y jóvenes^9^. Es en la Unidad Básica de Salud (UBS), a través de la capilaridad del SUS, donde se encuentra el mayor potencial para un cuidado adecuado de la salud de adolescentes y jóvenes. En sus propios territorios, en contacto con su vida cotidiana, su contexto sociocultural y sanitario, en convivencia longitudinal y de manera integrada con otros recursos y dispositivos sociales de la región, especialmente la escuela, se encuentran las mejores oportunidades para desarrollar capacidades de respuesta que permitan un cuidado efectivamente integral de la salud de adolescentes y jóvenes.

No obstante, la concreción de este potencial ha representado desafíos^10^^,^^11^^,^^12^. En el contexto actual, tras 37 años de existencia del SUS, ¿están los servicios logrando brindar una atención a la salud de adolescentes y jóvenes verdaderamente integral en el cotidiano de trabajo de las UBS? ¿Se encuentran los equipos de salud sensibilizados, preparados y dotados de suficientes recursos conceptuales, técnicos y materiales para llevar a cabo este trabajo? Con el propósito de contribuir a la respuesta de estas cuestiones, el presente estudio tiene como objetivo identificar aquellos aspectos del trabajo realizado en unidades básicas de salud del estado de São Paulo, Brasil, que los profesionales consideran como facilitadores u obstáculos para la producción de un cuidado integral a la salud de adolescentes y jóvenes.

## MÉTODO

El presente estudio es uno de los componentes de un proyecto más amplio sobre vulnerabilidades de adolescentes y jóvenes ante las ITS/VIH y la violencia de parejas íntimas, cuyo objetivo es evaluar la factibilidad, aceptabilidad y sostenibilidad de las prácticas de promoción de la salud y prevención de daños a la salud desde la perspectiva de los derechos humanos. Basado en el marco conceptual de la vulnerabilidad y derechos humanos^13^, el proyecto en curso se viene desarrollando desde el segundo semestre de 2019 en escuelas de enseñanza media en tres municipios del estado de São Paulo: São Paulo, Santos y Sorocaba.

A partir de las temáticas de salud sexual y reproductiva y violencia, pero integrando progresivamente otras condiciones sindémicas intervinientes a lo largo del proyecto, como el sufrimiento psicosocial, dificultades escolares, la pandemia de covid-19, el dengue y la mpox, se ha desarrollado un modelo denominado “prevención integral”.

Además de proponer un enfoque integrador en el diagnóstico de vulnerabilidades y en las formas de responder a ellas, el modelo en análisis prevé articular las acciones del entorno escolar con la Unidad Básica de Salud (UBS) en el propio territorio donde se ubica la escuela. Con este propósito, uno de los ejes de la investigación se dedicó al diagnóstico de las posibilidades y limitaciones de estas UBS para el trabajo con adolescentes y jóvenes desde la perspectiva de la integralidad, considerando tanto las acciones que efectivamente realizaban en su cotidianidad, como las sinergias potenciales a ser construidas con el trabajo en curso en las escuelas.

Para ello, se llevó a cabo un estudio cualitativo que, si bien incluyó observación del cotidiano de trabajo y entrevistas semiestructuradas a tanto a profesionales de salud como a personas usuarias de seis unidades de salud de los tres municipios participantes en la investigación, en el presente artículo solo se trabajan las entrevistas con las y los profesionales, contrastándolas con aspectos observados en los servicios.

 Se incluyeron territorios periféricos con patrones socioeconómicos que variaban desde situaciones de alta vulnerabilidad (favelas) hasta barrios de clase media baja y media, segmentos en los que el acceso, la equidad y la integralidad de la atención ofrecida en el territorio son prioritarios, dada la necesidad de acceso al sistema público y la fuerte dependencia del SUS para la promoción, protección y recuperación de la salud.

La investigación de campo se llevó a cabo entre enero y noviembre de 2023. Tanto las observaciones como las entrevistas fueron realizadas por el equipo de investigación, compuesto por docentes e investigadores de diversas universidades, con un enfoque multidisciplinar (medicina, psicología, ciencias sociales, terapia ocupacional, educación física y nutrición), siguiendo un guion orientado a la exploración de cuatro ejes de análisis construidos a partir de la *teoría del proceso de trabajo en salud* (TPTS)^14^.

Desarrollada por Ricardo Bruno Mendes Gonçalves, uno de los principales formuladores de los conceptos que orientaron la construcción del campo de la salud colectiva^15^, la teoría del proceso de trabajo en salud parte del materialismo histórico, en diálogo con pensadores de una epistemología crítica (Canguilhem, Foucault), para romper con los enfoques idealistas y ahistóricos que tratan las prácticas de salud como una mera aplicación de conocimientos científicos en técnicas orientadas a la restitución de la salud. En contraste, la teoría del proceso de trabajo en salud busca explorar la consustancialidad de las tecnologías de la salud con los procesos sociales y políticos de los contextos en los que se desarrollan y aplican.

Al entender las prácticas de salud como trabajo, la teoría del proceso de trabajo en salud permite identificar e interrelacionar analíticamente los componentes básicos de sus tecnologías, caracterizando el sentido social y político de sus diversas configuraciones en diferentes contextos locales y épocas. Dichos componentes pueden describirse sintéticamente como:

1. Objeto: el sustrato sobre el cual intervendrá el proceso de transformación operado por el trabajo;
2. Producto: el resultado que se busca alcanzar con ese proceso, de acuerdo con sus finalidades;
3. Instrumentos: recursos materiales (artefactos, instituciones) e inmateriales (técnicas, saberes) utilizados para alcanzar las finalidades del trabajo;
4. Agentes: los sujetos que conciben y/o ejecutan los procesos de trabajo en sus diferentes etapas.

Desde esta perspectiva, no es posible comprender adecuadamente estos componentes y sus interconexiones sin considerar las necesidades de salud que se constituyen social e históricamente en los contextos en los que operan las tecnologías, así como los aspectos políticos que median las relaciones entre quienes formulan, operan y reciben sus productos.

Basándose en la teoría del proceso de trabajo en salud, el presente estudio busca abordar la integralidad y el desafío de su operacionalización en los servicios de atención desde cuatro perspectivas analíticas. En primer lugar, es necesario atender a qué *necesidades* orientan el trabajo que efectivamente se lleva a cabo. Tanto los profesionales como los usuarios de los servicios de salud conciben de alguna manera lo que debe lograrse cuando proponen o reciben algún tipo de cuidado en salud y, por lo tanto, aquello que se busca transformar (en el cuerpo, en el entorno, en el comportamiento, etc.).

En este sentido, al concebir necesidades, se conciben de manera indisoluble las *finalidades* del trabajo, es decir, lo que se pretende alcanzar como resultado de este. Para ello, es necesario movilizar una serie de recursos materiales e inmateriales que permitan concretar las finalidades del trabajo y que se organizan, estabilizan y reproducen en forma de tecnologías (clínicas, preventivas, rehabilitadoras, promotoras de la salud).

Con los procesos sociales y tecnocientíficos de división del trabajo, el control y manejo de las tecnologías materiales e inmateriales no están bajo la supervisión de un único agente ni se limitan a un solo espacio de trabajo, sino que se distribuyen entre diversas experticias profesionales y sectores responsables de su aplicación. En este sentido, la atención a la salud, y más aún cuando está orientada hacia la integralidad, requerirá de *articulaciones*, es decir, demandará una mayor integración interdisciplinaria, multiprofesional e intersectorial.

Como consecuencia, para que todos estos diferentes operadores del trabajo puedan integrar sus dominios de experticia y competencias con el fin de ponerlas al servicio de los destinatarios de las acciones de salud, serán de fundamental importancia las *interacciones* entre estos diversos sujetos, tanto entre profesionales entre sí como, especialmente, entre profesionales y las personas destinatarias del cuidado.

Sobre la base de estas cuatro perspectivas para el análisis de las prácticas de salud se definieron los cuatro ejes analíticos que orientan el presente estudio. En la Tabla 1, se sintetizan, para cada eje, las preguntas orientadoras que guiaron las entrevistas y observaciones en el servicio, así como su interpretación.

**Tabla 1:** Ejes analíticos basados en la integralidad de los procesos de trabajo.

Necesidades	¿Cuáles son las necesidades de salud identificadas? ¿Cómo se comprenden y valoran? ¿Cómo se abordan?
Finalidades	¿Qué acciones de promoción de la salud, prevención, tratamiento y rehabilitación se ofrecen, dentro y fuera de la unidad, y cómo se integran (o no) a proyectos de cuidados sensibles a la singularidad del cuidado y la promoción de la salud de los jóvenes?
Articulaciones	¿De qué modo los diferentes saberes y técnicas de las diversas disciplinas y profesionales de la salud se movilizan e integran para la comprensión de las necesidades de los jóvenes y en las acciones propuestas para responder a ellas, ya sea en el ámbito de la salud o en otros sectores como educación, cultura, justicia y bienestar social? ¿Qué diferentes instituciones están involucradas en estas respuestas?
Interacciones	¿Cómo es la interacción entre profesionales y las personas jóvenes que consultan las UBS? ¿Existe empatía, una actitud respetuosa y atención a la diversidad (en términos de género, raza, religión, cultura, edad, escolaridad y orientación sexual)? ¿Se consideran sus singularidades? ¿Se toma en cuenta la perspectiva de derechos humanos como referencia para la identificación de vulnerabilidades y la propuesta de cuidados?

Fuente: Elaboración propia.

El acceso a los servicios y a los profesionales y agentes comunitarios de salud fue negociado entre las instituciones académicas responsables de la investigación y las instituciones responsables de la atención a la salud, tanto a nivel central de las gestiones municipales como a nivel local de las unidades. No se predeterminó el número de profesionales entrevistados, ya que esto dependía de la estructura y del modo de organización del trabajo en cada unidad estudiada. El objetivo fue conformar un panel lo más representativo posible de los diferentes perfiles profesionales involucrados en los procesos de trabajo desarrollados en cada unidad.

Se priorizó la inclusión en las entrevistas de, al menos, la persona a cargo de la gestión de la unidad básica de salud, un o una profesional de medicina, un o una profesional de enfermería y agentes comunitarios de salud, debido a sus posiciones estratégicas en la definición y operación de los procesos de trabajo. No obstante, también se incluyó a una gama más amplia de profesionales, como se observa en la Tabla 2.

**Tabla 2:** Perfil de los servicios, profesionales entrevistados y aspectos observados. Municipios de São Paulo, Santos y Sorocaba, estado de São Paulo, Brasil, 2023.

Número de servicio participante	Servicios de salud			Personas entrevistadas			Observaciones
	Tipo	Gestión	Cantidad de equipos ESF	Especialidad	Género	Años al momento de la entrevista	
Servicio 1 (S1)	Unidad Básica de Salud	Organización Social de Salud	6	Enfermera responsable	Mujer	38	Unidad Básica de Salud: recepción, acogimiento, salas de espera, reuniones (apoyo matricial*, Núcleo de Prevención a la Violencia), actividades en grupo. Servicios y organizaciones del territorio: ONG “Casa do Zezinho” (institución que ofrece refugio durante el día, en períodos extraescolares, a niñas, niños y adolescentes en situación de vulnerabilidad social en materia de alimentación, higiene, atención a la salud) y el Servicio de Acogimiento Institucional para Niñas, Niños y Adolescentes (SAICA).
				Enfermera 1	Mujer	29	
				Enfermera 2	Mujer	42	
				Médica responsable	Mujer	49	
				Médico 1	Varón	26	
				Médico 2	Varón	41	
				Profesional de educación física	Varón	30	
				Nutricionista	Mujer	34	
				Psicóloga	Mujer	29	
				Fisioterapeuta	Mujer	42	
				Dentista responsable	Mujer	44	
				ACS 1	Varón	27	
				ACS 2	Varón	43	
				Gestor	Varón	-	
Servicio 2 (S2)	Unidad Básica de Salud	Organización Social de Salud	7	Terapista ocupacional	Mujer	39	Unidad Básica de Salud: aspectos generales de la unidad (área física, comodidad, clima institucional), consultas, visitas domiciliarias, reuniones (equipo técnico, enfermería, Núcleo de Prevención de la Violencia, equipos especializados en la atención a niños, niñas y adolescentes víctimas y/o testigos de violencia, apoyo matricial* con el Núcleo de Apoyo a la Salud de la Familia (NASF), en salud mental con los Centros de Atención Psicosocial (CAPS) y el Servicio de Acogimiento Institucional para Niñas, Niños y Adolescentes (SAICA), que tiene el objetivo de acoger y garantizar la protección integral a niñas, niños y adolescentes en situación de riesgo personal y social, y de abandono por parte de las famílias. Servicios y asociaciones del territorio: dos ONG; Centro para Niños, Niñas y Adolescentes; Centro para la Juventud; Programa Joven Aprendiz; Asociación Cultural (recreativa y deportiva).
				Psicólogo	Varón	37	
				Fonoaudióloga	Mujer	38	
				Profesional de educación física	Varón	43	
				Medicina	Varón	28	
				Médica	Mujer	-	
				Enfermera responsable	Mujer	-	
				Enfermera 1	Mujer	42	
				Enfermera 2	Mujer	-	
				ACS 1	Mujer	45	
				ACS 2	Mujer	30	
				ACS 3	Mujer	57	
				ACS 4	Mujer	49	
				ACS 5	Mujer	48	
				ACS 6	Mujer	44	
				ACS 7	Mujer	35	
				Gestión	Mujer	-	
Servicio 3 (S3)	Unidad Básica de Salud	Organización Social de Salud	7	Psicóloga 1	Mujer	-	Unidad Básica de Salud: recepción, salas de espera, acogimiento, grupos, apoyo matricial*, visitas domiciliarias. Servicios y asociaciones del territorio: foros del territorio, ONG y otros servicios como el Centro de Atención Psicosocial Infanto-Juvenil (CAPS IJ); Centro de Convivencia y Cooperativa; Casa del Adolescente.
				Psicóloga 2	Mujer	44	
				Nutricionista	Mujer	46	
				Fonoaudióloga	Mujer	34	
				Fisioterapeuta	Mujer	37	
				Médica	Mujer	55	
				Médico	Varón	26	
				Ginecóloga	Mujer	32	
				Farmacéutica	Mujer	32	
				Enfermero	Varón	38	
				Enfermera 1	Mujer	41	
				Enfermera 2	Mujer	44	
				Odontóloga	Mujer	48	
				Asistente social 1	Mujer	50	
				Asistente social 2	Mujer	32	
				Protección ambiental	Mujer	35	
				ACS 1	Mujer	28	
				ACS 2	Mujer	39	
				Gestora	Mujer	44	
Servicio 4 (S4)	Unidad Básica de Salud tradicional	Organización Social de Salud	3	Enfermera	Mujer	59	Unidad Básica de Salud: recepción, sala de espera, grupo de adolescentes.
				Psicóloga	Mujer	28	
				Asistente social 1	Mujer	45	
				Asistente social 2	Mujer	38	
				Pediatra	Mujer	40	
				Gestora	Mujer	42	
Servicio 5 (S5)	Unidad de Salud de la Familia	Municipal	3	Psicólogo	Varón	54	Unidad de Salud de la Familia: recepción, acogimiento en salas de espera, reunión del equipo, visitas al territorio, visitas domiciliarias. Servicios y asociaciones del territorio: atención especializada para prenatal de alto riesgo.
				Enfermera	Mujer	41	
				Asistente social	Mujer	54	
				ACS	Mujer	49	
				Gestora	Mujer	41	
Servicio 6 (S6)	Unidad Básica de Salud tradicional	Municipal	0	Técnica en enfermería 1	Mujer	53	Unidad Básica de Salud: Sala de espera, recepción, farmacia, sala de curaciones, salas de enfermería, odontología, servicio de esterilización de instrumentos, consultorios (clínicos, pediatría, ginecología), comedor para trabajadores. Servicios y asociaciones del territorio: Centro de Atención Psicosocial Infanto-Juvenil (CAPS IJ).
				Técnica en enfermería 2	Mujer	51	
				Técnica en enfermería 3	Mujer	40	
				Técnica en enfermería 4	Mujer	60	
				Técnica en enfermería 5	Mujer	46	
				Enfermero	Varón	47	
				Enfermera	Mujer	53	
				Médica	Mujer	29	
				Pediatra	Mujer	46	
				Gestora	Mujer	44	
				Psicóloga 1 Caps	Mujer	26	
				Psicóloga 1 Caps	Mujer	31	

Fuente: Elaboración propia.

ESF = Estrategia de Salud de la Familia; ACS = Agentes comunitarios de salud.

*Apoyo matricial: actividad de discusión y seguimiento de casos complejos por profesionales de diferentes formaciones y/o especialidades.

Los servicios de salud incluidos en el estudio (Tabla 2) combinan diferentes modelos de gestión vigentes en el SUS en São Paulo, al igual que en gran parte de Brasil. Pueden ser de gestión directa, es decir, administrados por el propio municipio en el que se ubican, o de gestión indirecta, a cargo de organizaciones sociales de salud (OSS), entidades privadas responsables de la gestión y operación de las unidades de salud mediante contrato con los municipios, que transfieren los recursos de acuerdo con el cumplimiento de metas de desempeño establecidas.

Asimismo, las unidades se diferencian entre aquellas denominadas “tradicionales”, que operan con equipos convencionales (clínica médica, pediatría, obstetricia, etc.), generalmente con modelos de atención más dependientes de la demanda activa de las personas usuarias, y las unidades de *Estrategia de salud de la familia*, conformadas por equipos con medicina general, enfermería, técnicos y técnicas de enfermería y agentes comunitarios de salud, cuyo modelo de atención está más territorializado y basado en una interacción activa y continua con las familias y las instituciones de su área de adscripción^16^.

Las unidades de ambas modalidades asistenciales pueden, además, contar con la participación de equipos multiprofesionales más amplios (odontología, psicología, fisioterapia, terapia ocupacional, nutrición, educación física, etc.), según las diferentes necesidades y características de la red de servicios en cada región^17^.

En la Tabla 2 se presenta una síntesis del perfil de los servicios estudiados, así como de las entrevistas y observaciones de campo, junto con las respectivas designaciones de las personas informantes para identificar los extractos incluidos en el cuerpo del artículo.

Se realizaron 73 entrevistas semiestructuradas, en encuentros únicos, con una duración promedio de 40 minutos. Además, se realizó observaciones de servicio durante una semana tipo, es decir, se procuró observar actividades de interés realizadas en cada uno de los períodos de funcionamiento de las UBS, considerando sus diversas periodicidades. También se incluyeron observaciones en espacios como la sala de espera, los pasillos y el comedor, que formaron parte del material empírico. Las entrevistas fueron grabadas y transcritas por profesionales especializados, y posteriormente revisadas por el equipo de investigación. Asimismo, las grabaciones realizadas por las y los investigadores tras las observaciones de los servicios, junto con las notas de los cuadernos de campo, fueron transcritas y recopiladas. 

Todo este material, que conformó el corpus discursivo que constituye el material empírico del estudio, fue sometido a un tratamiento hermenéutico (comprensivo-interpretativo)^18^. Es decir, el marco de la integralidad del cuidado desde la perspectiva de la *vulnerabilidad y derechos humanos* y de la *teoría del proceso de trabajo en salud* fue tomado como la totalidad comprensiva de referencia que permitió construir e interpretar la discursividad producida en campo. Dicha interpretación, estructurada en torno a los cuatro ejes de análisis expuestos anteriormente, fue enriqueciendo a su vez esta totalidad de referencia, configurando un “círculo hermenéutico”, que permitió situar en un nuevo nivel la comprensión de los fenómenos estudiados^19^.

Se tomaron las precauciones necesarias para garantizar la confidencialidad y el anonimato de las personas participantes, así como para evitar situaciones que pudieran generarles incomodidad, tanto en el momento de realización de las entrevistas como en su registro escrito y almacenamiento. El protocolo de la investigación fue sometido y aprobado por el Comité de Ética de la institución sede del proyecto, el Instituto de Psicología de la Universidade de São Paulo (CAAE 00530918.9.0000.5561). Todas las personas participantes fueron informadas y firmaron los términos de consentimiento libre e informado**.** Tanto el material grabado como las transcripciones, identificados mediante códigos, se encuentran almacenados en un drive de acceso exclusivo para el equipo de investigación, estando disponibles para consulta de terceros únicamente bajo los principios de interés público y buena práctica científica, manteniendo las garantías de confidencialidad y anonimato.

## RESULTADOS Y DISCUSIÓN

### Necesidades

Como se indicó anteriormente, las necesidades planteadas para el trabajo en salud van siendo incorporadas y reproducidas en forma de tecnologías que se legitiman socialmente y, en este sentido, tienden a ser asumidas en abstracto como universales y absolutas, compartidas tanto por los agentes del trabajo como por sus beneficiarios en cualquier tiempo y lugar. Sin embargo, en la realidad vivida, las diversas experiencias cotidianas de los distintos sujetos generan diferencias en la manera en que dichas necesidades son comprendidas y valoradas, lo que provoca tensiones, fisuras y/o conformidades que impactan en el trabajo efectivamente realizado y sus posibilidades de transformación.

En el presente estudio, sin olvidar que existe algún grado de consenso social más amplio, nos enfocamos específicamente en lo que consideran las personas que trabajan en las unidades básicas estudiadas sobre las necesidades de salud de adolescentes y jóvenes, sus vulnerabilidades y demandas, así como lo que perciben acerca de cómo estos adolescentes y jóvenes comprenden dichas necesidades.

Al investigar las necesidades de adolescentes y jóvenes en los territorios de las UBS, la tendencia de las personas informantes fue catalogar las demandas hacia los servicios, observándose una referencia significativa a la búsqueda de métodos anticonceptivos; pruebas rápidas para el diagnóstico de ITS y embarazo tras situaciones de exposición y riesgo, un patrón común de utilización de estos grupos en los servicios de salud^9^. También se destacó el aumento de la demanda espontánea para la atención de molestias clínicas agudas y sufrimientos psicosociales, lo que corrobora investigaciones recientes sobre esta temática^20^.

Las cuestiones relacionadas con el consumo de alcohol y otras drogas emergieron como una demanda indirecta, generalmente atribuida al contexto familiar y a vulnerabilidades psicosociales. Asimismo, se observó un predominio de las demandas formuladas por mujeres, lo que refleja una barrera de acceso vinculada al género, fenómeno que también ha sido identificado en otros contextos latinoamericanos^21^.

“*A los chicos, en realidad, casi no los atiendo. No nos consultan. Así que tampoco tenemos nada dirigido a ellos…, pero es que no nos buscan. Son más bien las chicas*”. (S5_Enfermera)

Algunas de las personas entrevistadas destacaron lo que consideran un inicio precoz de la vida sexual de las niñas, entre los 12 y 14 años. La demanda por métodos anticonceptivos fue señalada como la principal motivación para la búsqueda de atención por parte de niñas cis, especialmente en el grupo de edad de 14 a 16 años. Entre los métodos más solicitados por adolescentes y jóvenes se encuentran el dispositivo intrauterino en forma de T (DIU de cobre, con una duración de hasta 10 años), el acetato de medroxiprogesterona (disponible en comprimidos y en solución inyectable con una duración de tres meses) y el implante anticonceptivo subdérmico de etonogestrel (ICS**,** con una duración de hasta tres años), debido a que son métodos de larga duración y menos susceptibles a errores o fallos en su uso.

El ICS es un método de prevención del embarazo de larga duración disponible en el SUS desde 2022, aunque con políticas de distribución variables según el municipio. Es un método que se ha vuelto popular entre las adolescentes y jóvenes, siendo evaluado por las y los profesionales como altamente eficaz. Según el Ministerio de Salud de Brasil^22^, respetando las particularidades municipales, se recomienda su oferta a mujeres cis en edad reproductiva entre 18 y 49 años, que se encuentren en situaciones de alta vulnerabilidad, como situación de calle, violencia, consumo abusivo de alcohol y otras drogas y/o inmunosupresión. 

No obstante, la demanda a veces excede este perfil y se menciona, incluso, que la preocupación por su uso no siempre proviene de la propia joven:

“…*hay algunas que se sientan ahí y ¡no dicen nada!* [Risas] *y ahí dicen ‘vengo a ponerme el chip’. Y la madre al lado, y es la madre quien habla, ¿sabés? Entonces, te das cuenta de que… que de alguna manera están siendo influenciadas a hacerlo*” (S3_Ginecóloga)

Aunque la información sobre embarazo varió entre los servicios, las y los profesionales, en general, enfatizan la preocupación por este aspecto, aunque de manera poco contextualizada en relación con la singularidad de los modos de vida. En uno de los relatos, se destaca el desajuste que a veces ocurre en la manera en que se interpretan las necesidades cuando se trata de salud sexual y reproductiva, lo que termina por convertirse en un obstáculo para una aproximación más integrada a los contextos y perspectivas de adolescentes y jóvenes:

“*...hay algo que considero muy marcante, que es cuando un adolescente elije, ELIJE, el momento de tener la primera relación sexual. Es su primer acto sexual y busca cuidarse ¿no? ... y cuando llega a la UBS y escucha de enfermeros, médicos, técnicos de enfermería que… es muy joven para hacer eso. Esa… esa falta de cuidado que los profesionales tienen, para mí, es lo peor. No están ahí para juzgar. Si el adolescente hizo la elección, nada va a modificar… la elección de él. Si es ese el momento, y fue a buscar… un método anticonceptivo para NO embarazar, acoja. ¡Acoja y listo!*”. (S5_Asistente social)

Las demandas psicosociales fueron ampliamente mencionadas por profesionales de diferentes áreas de formación: ideación/intentos de suicidio, ansiedad, autolesiones, miedos, tristeza, dificultad para dormir, nerviosismo, impaciencia, aislamiento social, diagnósticos de trastorno límite de la personalidad (*borderline*), esquizofrenia y depresión.

Algunas de las personas entrevistadas relacionan estas demandas con los determinantes sociales de la salud y con vulnerabilidades psicosociales. Factores como las relaciones con sus pares en la escuela (*bullying*) y con sus familiares, incluyendo la ausencia de diálogo, negligencia en los cuidados y falta de afectividad, violencias (físicas y/o psicológicas), la violencia en el territorio vinculada al narcotráfico en una de las áreas estudiadas, la inseguridad respecto al futuro, la pérdida de ingresos familiares, la fragilidad en la red de protección social, la falta de oportunidades tanto en el ámbito educativo como en la inserción en el mercado laboral, todo ello agravado por la experiencia de la pandemia, con sentimientos de pérdida, duelo y restricciones sociales, fueron patrones identificados en este estudio, en consonancia con diversas otras investigaciones en distintos contextos^23^^,^^24^.

“*...el sufrimiento mental durante la pandemia entre los adolescentes y jóvenes, que se quedaron en casa sin mucho contacto con personas de su edad, perdieron años de socialización, de aprendizaje de diversas habilidades, de experiencias. Esto les causó sufrimiento. El aumento en la demanda por atención en salud mental es una nueva pandemia que es consecuencia de la pandemia*”. (S3_Gestora)

Además de las cuestiones relacionadas con la salud sexual y reproductiva y la salud mental, también se señalaron demandas relativas a la autoimagen (problemas dermatológicos, reducción mamaria debido a repercusiones ortopédicas, estandarización corporal), al género (afirmación y transición), a las creencias y valores morales divergentes y a la nutrición.

En el servicio 1, la profesional nutricionista destacó necesidades relacionadas con la alimentación, como adolescentes con obesidad o con algún trastorno alimentario debido a limitaciones en la estructura familiar y financiera. Sin omitir la búsqueda de encuentros que privilegiaban un enfoque más educativo sobre la alimentación, que parecería indicar cierta visibilidad del cotidiano de adolescentes y jóvenes, se observa una mirada marcada por generalizaciones y por prácticas aún poco conectadas con la vida en el territorio.

Los marcadores sociales y sus aspectos interseccionales no suelen ser considerados en los enfoques educativos dirigidos a diferentes demandas:

“*La educación en salud es la base para la prevención.* [...] *Creo que esto* [refiriéndose a negligencias en el autocuidado] *está mucho más relacionado con la falta de información que con la edad, la raza, el sexo, la orientación o la religión*”. (S3_Enfermero)

Las preocupaciones sobre la vida escolar son un aspecto muy relevante para la integralidad del cuidado de adolescentes y jóvenes. Al respecto, los profesionales destacan demandas relacionadas con dificultades de aprendizaje, sociabilidad y desarrollo neuropsicomotor, aunque de manera no articulada con los procesos de trabajo propuestos por la unidad, sino más bien como demandas espontáneas:

“*…La escuela ha estado trayendo casos y hemos tratado de resolverlos. Algunos más graves, otros más leves. También está esta cuestión del autismo, que ha aumentado mucho; muchos fueron diagnosticados con autismo, otros con TDAH, otros, niños con hiperactividad. Y gracias a las profesoras, que también han hablado, y a la inspectora, que ha llevado estos casos a las reuniones...*” (S5_Agente Comunitario de Salud)

### Finalidades

En el desarrollo de acciones dirigidas a adolescentes y jóvenes en la atención primaria, uno de los desafíos fundamentales para los servicios se refiere a la transición de una atención individual hacia un cuidado más amplio y colectivo, basado en la promoción de la salud y la reducción de vulnerabilidades ante enfermedades. Los relatos y observaciones mostraron que existen algunas acciones en este sentido en las unidades, pero no específicamente dirigidas a este grupo, aunque algunas y algunos profesionales señalaron la importancia de iniciativas en esta perspectiva:

“*Nuestra preocupación en el trabajo con niños, niñas y adolescentes es transitar de este modelo de cuidado individual hacia posibilidades más amplias, que involucren la promoción de la salud, la prevención de daños y una serie de cuestiones que están surgiendo*”. (S5_Psicólogo)

A pesar de que la promoción de la salud y la prevención de enfermedades son nociones que fundamentan el trabajo en la atención primaria, distintos factores (como los modelos de tercerización de la gestión a través de contratos con las organizaciones sociales de salud, la reducción o ausencia de espacios formativos más críticos orientados a la educación permanente de los equipos y el subfinanciamiento del SUS) han contribuido significativamente al fortalecimiento de un enfoque preventivista, que privilegia la prescripción individualizadora de “cambio de estilos de vida”.

Desde esta perspectiva, destacamos que las finalidades predominantemente observadas en los procesos de trabajo en las unidades de salud estudiadas están relacionadas con la prescripción de métodos anticonceptivos y la realización de exámenes, así como con la atención puntual de demandas, como se señaló en la sección anterior.

Las actividades en grupos están entre las estrategias más mencionadas por los profesionales entrevistados para la acogida y el seguimiento de adolescentes y jóvenes. Sin embargo, se señalaron diversas dificultades operativas que obstaculizan estas actividades, y muchas de las personas entrevistadas indicaron que no existen programas específicos para este grupo en los servicios. Algunos y algunas profesionales identifican la necesidad de incorporar otras estrategias para el abordaje de la prevención y promoción de la salud, con la perspectiva de que un grupo para adolescentes y jóvenes pueda constituirse como un espacio seguro y atractivo para la orientación en salud, ya que, como han demostrado otros estudios, la adherencia de adolescentes y jóvenes a actividades educativas en grupo suele ser baja^25^.

En relación con la salud sexual y reproductiva, se subrayó la necesidad de desarrollar actividades orientadas a fomentar una mirada crítica sobre la información que los adolescentes y jóvenes acceden en Internet. Entre los temas señalados por las y los profesionales, como asociados a la desinformación de este grupo, se encuentra el uso ampliamente difundido del método anticonceptivo ICS**:**

“*La información va circulando* […] *la amiga se lo puso, no sé quién más se lo puso, y ellas también lo quieren.* […] *La mayoría es así. Difícilmente viene alguien sin saber qué es. La mayoría ya lo sabe. Ellas lo llaman ‘el chip’*.” (S3_Ginecóloga)

Las actividades desarrolladas en el contexto escolar están, en su mayoría, relacionadas con campañas, principalmente de vacunación, llevadas a cabo por profesionales de enfermería en el marco bastante desafiante^26^ como es el Programa Salud en la Escuela, establecido dentro de las políticas de salud y educación con el propósito de promover la salud y la educación integral en las escuelas bajo la responsabilidad de las UBS.

“*Hay cosas para las que actuamos de manera activa.* [...] *Recibimos solicitudes para la búsqueda activa de vacunación, por ejemplo, revisar la cartilla, hacer los DVA* [Declaración de Vacunación Actualizada]. *Durante la pandemia realizamos la toma de serología de los docentes y encuestas serológicas*”. (S1_Enfermera RT)

Además, se mencionaron experiencias previas en el entorno escolar que ofrecen indicios sobre la efectividad de las acciones integradas con el sector salud, así como sobre sus limitaciones y posibles caminos en cuanto al formato y contenido de dichas acciones.

“*Por la experiencia que tengo, hay muchas cosas que podemos hacer, pero no tengo ninguna escuela en mi área. En otro servicio en el que trabajábamos, realizábamos tratamiento antiparasitario, charlas sobre pediculosis, sarna. Con niños mayores, hablábamos sobre embarazo en la adolescencia. Este grupo de charlas también abordaba temas puntuales de interés para los adolescentes. ¡Son maravillosos! También brindábamos cobertura médica en la jornada deportiva de la escuela*…” (S1_Médico 2)

### Articulaciones

Un primer nivel de articulación para la implementación de un cuidado integral se refiere al propio equipo, a su capacidad de reconocer e integrar los diferentes saberes y competencias técnicas para una adecuada interpretación y respuesta a las necesidades de salud. En este sentido, es importante destacar dos aspectos que obstaculizan esta articulación: las prioridades de la gestión, que favorecen poco la conformación y el fortalecimiento ético-político de los equipos, dada la reducción o incluso la ausencia de espacios colectivos para analizar y planificar el trabajo, y la falta de prioridad en la contratación de equipos completos con regímenes de trabajo integral, lo que resulta en profesionales que trabajan con jornadas reducidas y/o se reparten entre más de una unidad de salud^27^.

Estos movimientos guardan una estrecha relación con la fragmentación de la mirada que aún predomina en los servicios de salud y con la reducción progresiva de espacios de diálogo y acuerdos que permitan a los equipos problematizar las acciones desarrolladas o propuestas y las formas de sostenerlas. Desde la perspectiva de un cuidado integral, esto requeriría también la participación activa de adolescentes y jóvenes.

“*Tiene que haber más comunicación entre los profesionales y alinear, conectar la información. Tal vez crear un proyecto singular para que tenga sentido en ese contexto. Si tuviéramos más tiempo para reuniones de apoyo matricial, la atención a este público, o a cualquier paciente, sería más efectiva, ¿no?* [...] *La agenda de todos está sobrecargada. Antes teníamos dos reuniones de matriciamiento al mes para cada equipo. Ahora es una cada 15 días para todos los equipos. Antes los agentes de salud también participaban. Ahora solo está la enfermera. Esa riqueza de detalles que los agentes de salud aportaban, la hemos perdido. A veces conocen a la persona desde que nació*”. (S1_Nutricionista)

En relación con las articulaciones entre los diferentes niveles de atención a la salud y entre distintos sectores de actividad, se reconoce la existencia de recursos y, como en otros contextos, su importancia. Sin embargo, en algunos casos, se identifican limitaciones para el reconocimiento de alternativas ya existentes y/o dificultades en su implementación efectiva:

“*La integración que existe y el tamaño de la red municipal, la cantidad de servicios... solo es cuestión de llegar al lugar correcto, y ahí se resolverá. Muchas veces el problema no es la red, muchas veces es el profesional. La red está ahí, pero el profesional no sabe cómo utilizarla.* [...] *Es un desconocimiento de la red, o una falta de empatía, o una falta de voluntad para actuar*”. (S1_Médico 2)

“*En la reunión de red, participan todos los servicios que están involucrados con esa persona... asiste la escuela, el consejo tutelar, si es el caso, el CREAS* [Centro de Referencia Especializado en Asistencia Social], *el CRAS* [Centro de Referencia de Asistencia Social]... *convocamos a toda la red que está involucrada en esa situación*” (S5_Gestora)

En relación con la articulación entre la UBS y la escuela, una modalidad de articulación intersectorial que se ha demostrado fundamental para una atención integral a adolescentes y jóvenes y sus principales demandas^28^^,^^29^^,^^30^, las personas entrevistadas confirman que este es un camino potente a desarrollar, destacando el hecho de que el adolescente “*ya está en la escuela*”, “*está en su grupo*”, lo que puede favorecer encuentros y lecturas más enriquecedoras de sus necesidades. No obstante, advierten que el conocimiento sobre las escuelas del territorio, en general, es limitado, aunque existen acciones desarrolladas en colaboración, ya sea por demandas expresamente planteadas por la escuela o por iniciativa de la UBS:

“*Las escuelas forman parte de nuestros equipamientos. Algunas son más abiertas que otras. Hay cosas para las que actuamos de manera activa... Ahora retomamos el PSE* [Programa Salud en la Escuela]. *Algunos temas los sugiere el PSE. Nuestra APA, la Agente de Protección Ambiental, tiene un trabajo en la EMEI* [Escuela Municipal de Educación Infantil]”. (S1_Enfermera responsable)

Los equipos identifican, sin embargo, algunos obstáculos importantes para esta articulación:

1) Alto número de escuelas y pacientes en el área de adscripción:

“*La escuela, en sí, trabaja más en la fiscalización de las cartillas de vacunación, porque no logramos abarcar todo, son demasiadas escuelas, tenemos demasiados pacientes* [...] *Por más que queramos hacer algo más, no nos queda tiempo*”. (S3_ACS 1)

2) Falta de articulación para el planeamiento conjunto entre salud y educación, tanto a nivel municipal como estatal:

“*La municipalidad no tiene una asociación directa con las escuelas estatales, trabaja más con las municipales. Entonces, nuestra relación es más bien de vínculo con las directoras* [...] *Una dificultad es esa falta de acuerdo entre la municipalidad y el estado. No se comunican y eso nos impacta mucho ¿no? A veces hay acciones... Tenemos que hacer el PSE* [Programa Salud Escolar] *y yo dirijo al dentista, al área de odontología, a enfermería para hacer antropometría, actividades*. […] *pero solo puedo enviar a las escuelas municipales. Son las únicas que están registradas*”. (S4_Gestora)

3) Ausencia de integración intrasectorial (salud municipal y estatal):

“*No hay coordinación. El estado no se comunica con el municipio. Si tengo un paciente internado en el* [hospital estatal], *nadie envía un correo a la UBS* [informando]”. (S3_Médica)

4) Organización del trabajo centrada en la atención individual:

“*Creo que las escuelas abrirían sus puertas, pero el problema, volvemos a lo mismo, es la cuestión de la agenda, de tener un horario y contar con los profesionales*. [...] *Fuera del servicio de atención, lo que tenemos es la visita domiciliaria, dirigida a los pacientes en cama. Nosotros, los médicos, no tenemos ninguna otra actividad externa, nada. Todavía participo en un grupo de cesación del tabaquismo, pero casi no puedo asistir porque choca con mi agenda*”. (S1_Médica responsable)

También se identificó el riesgo de una cierta imposición en la participación de adolescentes y jóvenes en la integración UBS-escuela.

“*Es diferente cuando invito a un adolescente a un grupo en la* UBS*, tal vez venga, tal vez no: ‘Qué pereza ir a la* UBS *a escuchar a alguien hablar’... Es distinto si voy a la escuela, quiera o no, va a escuchar algo, porque estará medio obligado a participar. Algo lograremos alcanzar*”. (S1_Médica responsable)

Otro obstáculo para la integración UBS-escuela está relacionado con el currículo y cronogramas poco flexibles en las escuelas, especialmente considerando que, como se ha enfatizado, adolescentes y jóvenes, así como las propias escuelas, buscan los servicios de salud de manera episódica. Además, cuando existen colaboraciones, estas se restringen, más allá de la vacunación, a la realización de charlas.

“…*una charla de una hora no va a cambiar los hábitos para toda la vida. Es necesario educar para que las personas también sean capaces de difundir esa información*. […] *Cuando nos acercamos a las escuelas para formar un grupo, siempre surgió la cuestión de que no había espacio en el calendario. El horario de clases ya estaba cerrado*”. (S3_Gestora)

En el marco del contexto actual de avance del conservadurismo, este escenario en las escuelas remite a una proscripción del debate sobre temas considerados sensibles para las familias de las y los estudiantes (especialmente sexualidad y género), así como a la dificultad de incluir asuntos de interés para los jóvenes:

“*Aquello que se sale del currículo es tratado como problemático, hay una tendencia a evitar el tema. Cualquier enfoque que vaya más allá de lo biológico, de lo ‘higienizado’, genera conflictos*”. (S3_Gestora)

“*La escuela reconoce la necesidad de hablar sobre sexualidad, pero los padres no quieren. Entonces, terminan limitando el tema. Una vez preparé una charla sobre el cuerpo humano porque no podía hablar sobre sexo. Y, en ese contexto, intentas de manera muy sutil orientar, porque son adolescentes que hacen preguntas capciosas. Los padres no ven la necesidad, ¡pero ellos tienen mil dudas!*” (S2_Enfermera 1)

La Tabla 3 presenta una síntesis de los factores facilitadores y los desafíos en relación con la articulación UBS-escuela. Las articulaciones intersectoriales, interinstitucionales y comunitarias amplias emergen en el escenario contemporáneo como componentes fundamentales para la implementación de la integralidad del cuidado de la salud de adolescentes y jóvenes en diversos contextos de América^5^^,^^28^^,^^30^.

**Tabla 3:** Facilitadores y obstáculos para la integración salud-escuela, según las y los profesionales entrevistados. Municipios de São Paulo, Santos y Sorocaba; estado de São Paulo, Brasil, 2023.

Facilitadores	Obstáculos
La realización de acciones de salud en la escuela puede ser facilitada por el hecho de que es un espacio de confianza para las y los adolescentes.	Agendas de trabajo sobrecargadas (tema más citado).
Las acciones en la escuela pueden volverse más efectivas en la medida en que los profesionales se acercan a las y los adolescentes.	Número insuficiente de profesionales de la salud para el desarrollo de acciones/programas en las escuelas.
Un abordaje más colectivo de los temas relacionados con la vida de adolescentes y jóvenes puede verse favorecida por el conocimiento de las necesidades vividas en los territorios.	Horario de realización de las actividades en las escuelas en relación con la carga horaria de las y los profesionales de salud en las UBS, especialmente las personas que integran los equipos multiprofesionales (hasta las 13h o que trabajan en dos UBS).
Escuelas y UBS tienen las puertas abiertas (acceso entre los espacios), lo que facilita la construcción de diálogos e iniciativas conjuntas.	Escasa articulación para el desarrollo de propuestas y acuerdos entre la UBS y la escuela: el trabajo en la UBS está vinculado al ámbito municipal, mientras que las escuelas dirigidas a adolescentes y jóvenes dependen del ámbito estatal.

Fuente: Elaboración propia.

En nuestro estudio, además de la escuela, se mencionaron otros recursos institucionales en los territorios que trabajan con jóvenes y que pueden ser buenos aliados de las UBS**.** Equipos como los Servicios de Acogimiento Institucional para Niños, Niñas y Adolescentes (SAICAS), los Centros para Niños, Niñas y Adolescentes (CCA), los Centros de la Juventud (CJ) y diversas organizaciones no gubernamentales (ONG) desarrollan actividades culturales, deportivas y de formación profesional, que pueden ser fundamentales para la articulación de acciones de salud y la ampliación de la red de promoción de la salud para adolescentes y jóvenes.

“[la ONG X] *es lindo* [...] *porque todo es de primera, ¿no?* [...] *Y ahí tienen actividades de gastronomía e informática, además de otras actividades. También trabajan un poco el proyecto de vida*”. (S2_Psicólogo)

“*Teníamos un grupo de adolescentes en la radio* [comunitaria]. […] *¡Se llenaba de adolescentes! Porque era una radio, había música, rap, samba, funk. Tenían un taller y hacíamos grupos con ellos ahí, en una sala entera dedicada al grafiti*. […] [Los gestores de la radio] *encontraron la manera de atraer a estos adolescentes y hacer que se responsabilizaran de cada actividad*”. (S3_Enfermera 1)

### Interacciones

El diálogo entre los profesionales de salud y las personas destinatarias de sus acciones es una condición necesaria para que las finalidades y los medios del cuidado estén en sintonía con los aspectos singulares de las necesidades que lo justifican y, por lo tanto, para que la integralidad pueda ser efectivamente incorporada en los procesos de trabajo^31^.

Las entrevistas evidenciaron la percepción de que las y los adolescentes y jóvenes forman parte de un público considerado de difícil interacción, que asiste poco a los servicios de salud y que no muestra interés en las acciones de prevención y educación en salud. En este contexto, el testimonio de una profesional ofrece pistas importantes sobre la necesidad de que los equipos construyan otras formas de interacción con adolescentes y jóvenes:

“*Creo que deberíamos reflexionar un poco más sobre cómo la UBS puede ser más acogedora para los adolescentes. Ellos vienen aquí en la medida en que lo necesitan o cuando los traen sus padres. ¿Cuánto logramos, como equipo, capacitarnos mejor para atender sin usar nuestra propia historia de vida, nuestras creencias individuales? He visto muchas situaciones que se juzga. En un grupo de gestantes en el que había adolescentes, un dentista llegó para hacer orientación y preguntó: ‘¿Cuántos años tienen?’. Ellas respondieron y él dijo: ‘Puf, es temprano para tener hijos ¿no?’. ¿Cómo va a volver esa adolescente a esta UBS? Necesitamos reflexionar más sobre esto y ofrecer un poco más de acogimiento para ellos, quizás en la forma en que ingresan al servicio... Hoy tenemos un grupo que funciona por la mañana. ¿Y los adolescentes que estudian en otro turno? No estamos cubriendo todos los horarios. Tenemos que construir esto un poco más junto con ellos*”. (S2_Terapeuta Ocupacional)

En esta misma línea, una enfermera subrayó la necesidad de desarrollar un trabajo con el equipo técnico, junto con un mayor incentivo para crear espacios que favorezcan otras formas de interacción, diálogo, acceso y acogida para adolescentes y jóvenes, ya que:

“*Los profesionales de salud no reciben mucha formación sobre esto en la universidad, no llegan con ese enfoque sobre cómo acercarse sin juzgar*”. (S1_Enfermera Resp.)

Cuando se les preguntó sobre aspectos que pueden mejorarse en el cuidado de adolescentes y jóvenes y los desafíos enfrentados, las y los profesionales señalaron distintos elementos, incluyendo el proceso de trabajo. Se hizo referencia a la necesidad de una percepción más amplia sobre las y los adolescentes y jóvenes que buscan el servicio de salud, con el objetivo de comprender cómo sus necesidades y demandas se manifiestan y repercuten en sus vidas, superando el modelo de demanda-conducta.

“*A veces, si un adolescente va a la sala de vacunación, ¿el profesional que está ahí tiene esa mirada? Tal vez podríamos aprovechar estas situaciones que ya atraen a este público de alguna manera. La vacunación es una de ellas, porque debe estar al día, hay un vínculo con la escuela, ¿no? Sería una herramienta capacitar a estos profesionales de la sala de vacunación para que, cuando llegue un adolescente, puedan preguntar: ‘¿Cuándo fue la última vez que tuviste una consulta?’, con el fin de intentar atraer a este adolescente, de alguna forma, a nuestro servicio*”. (S2_Médica)

Esto implica sensibilidad y apertura a un diálogo en el que no se juzgue a las y los adolescentes y jóvenes que pueden llegar, por ejemplo, con una solicitud de consulta de psicología, una demanda escolar, una petición de prueba de embarazo o una acción de vacunación. Así, pueden generarse movimientos (individuales y colectivos) para interrogar: ¿qué aproximaciones y diálogos pueden construirse para un cuidado más integral?

“*Un ejemplo de esto: atendí a un joven, creo que de mi edad, y se sentía muy triste con la vida. Entonces, la consulta toma un poco más de tiempo, ¿no? Y terminamos atrasando toda la agenda* […]. *Hablas con él, le explicas, le ofreces medidas, apoyo, porque ellos creen que el médico está aquí solo para medicar, recetar y derivar. Cuando hacemos esto, les mostramos que no estamos aquí solo para eso. Estamos aquí para acompañarlo, para que tenga un apoyo. A todos los pacientes que atiendo, les recomiendo un retorno. Porque así ven que me intereso por ellos*”. (S3_Médica)

Además de la necesidad de mayor apertura y de otras formas de acercamiento con adolescentes y jóvenes, también se enfatizó la importancia de la interacción con las familias para facilitar la identificación de sus demandas y la construcción de un trabajo más integrado con la comunidad, especialmente con el apoyo de las y los agentes comunitarios de salud.

“*No conocemos mucho la demanda de los otros profesionales, ¿no? Es un poco complicado hablar de eso. Por ejemplo, ellos* [los agentes comunitarios de salud] *hacen una visita por mes... podríamos estar un poco más cerca. Creo que, si logramos fortalecer esa base, con los agentes de salud, que son primordiales para nosotros, más cercanos a la familia, podríamos captar mejor parte de la demanda de los adolescentes*”. (S1_Profesional de Educación Física)

Los relatos de las y los agentes comunitarios de salud permitieron escuchar movimientos más dinámicos, que exceden los muros de las UBS, y que pueden abrir caminos para la construcción de confianza entre adolescentes y jóvenes, facilitando su acercamiento al trabajo realizado en atención primaria.

“*Creo que nosotros, los agentes de salud, somos mucho más buscados que la unidad de salud en sí, más que la llegada del adolescente hasta aquí* […]. *Hubo un caso de un adolescente que tenía el pene inflamado, con secreción, y no habló con su madre. Me mostró una foto y tuvo la libertad de enseñármela. Vino hasta aquí* [a la UBS] *por orientación mía. Sabe que voy a su casa, sabe que trabajo en el centro de salud. De repente, sintió más confianza, un vínculo mayor*”. (S3_ACS)

“*Como todavía soy nuevo, dependo mucho de la historia clínica para conocer a los pacientes en la primera consulta. Generalmente, quienes traen esta información son las agentes de salud... Creo que ellas son la principal fuente de información sobre los pacientes en atención primaria*”. (S1_Médico 1)

En cuanto a los aspectos del proceso de trabajo que repercuten en la producción del cuidado de adolescentes y jóvenes, la ya mencionada centralidad en el cumplimiento de metas basadas en indicadores cuantitativos de atenciones individuales ha empobrecido la interacción con los espacios comunitarios, limitando la comprensión de las necesidades de salud a partir de interacciones más dinámicas en los territorios.

Un ejemplo ilustrativo de este distanciamiento se constató al intentar obtener información más detallada sobre el número de adolescentes y jóvenes registrados en la UBS, o sobre las características de esta población en su territorio de cobertura. Las respuestas más comunes fueron que los datos eran escasos o que “*no se disponía de ellos en el momento*”. También se evidenció la afirmación recurrente de las personas entrevistadas de que los datos e información utilizados para conocer a las y los adolescentes y jóvenes se limitaban a la historia clínica.

Este distanciamiento sin duda dificulta la comprensión de las vulnerabilidades en salud y su relación con la promoción y protección de los derechos de adolescentes y jóvenes en los territorios estudiados, lo que se refleja en la casi inexistente referencia a estos aspectos en las entrevistas y en las actividades observadas. A su vez, esto compromete la aproximación al ideal de integralidad del cuidado.

Estos hallazgos coinciden con estudios previos que evidenciaron que la forma en que se organiza el acceso a las UBS (programación de citas, tiempo de espera para la consulta, filas para la atención de urgencias) no favorece la llegada de adolescentes y jóvenes a los servicios ni la búsqueda de cuidados en salud^32^. El escaso tiempo de atención, la falta de empatía de las y los profesionales y el predominio de prácticas biomédicas también contribuyen a la falta de vinculación de adolescentes y jóvenes con los servicios de salud^32^^,^^33^^,^^34^^,^^35^.

Las observaciones de las y los profesionales coinciden con hallazgos de algunos estudios que señalan la ausencia de una mirada diferenciada hacia las y los adolescentes y jóvenes, la persistencia de visiones negativas, prejuiciosas y adultocéntricas, así como de prácticas que no favorecen la autonomía, el autocuidado ni una actitud preventiva en adolescentes y jóvenes, lo que debilita la garantía de sus derechos^33^^,^^35^^,^^36^.

Las interacciones entre las y los profesionales también se ven afectadas en este modelo de trabajo, donde las actividades de los equipos tienden a fragmentarse y aislarse. Aunque se identificó una valoración de las consultas compartidas y de las reuniones con el equipo multiprofesional, las actividades conjuntas dependen de arreglos informales basados en iniciativas individuales y en posibilidades situacionales, como la coincidencia de agendas y compromisos.

“*De alguna manera, lo creamos individualmente y se transmite de manera muy informal. Es más bien una conversación de pasillo, llamar a una colega para hablar. O decir: ‘entra aquí en la sala, únete a mi grupo’*. […] *Tratamos de hacerlo dentro de lo posible. Por ejemplo, si un paciente falta o si veo un espacio en la agenda, me siento con otro profesional y discutimos el caso, o si tengo un paciente que necesita otra especialidad, busco al profesional y converso con él. No es algo muy rígido: ‘Si no hay horario, no se hace*’”. (S3_Nutricionista)

## CONSIDERACIONES FINALES

Nuestros resultados muestran cuán desafiante es la implementación concreta del principio abstracto de la integralidad en la atención a la salud, particularmente en lo que se refiere al cuidado de adolescentes y jóvenes en atención primaria, lo que corrobora otras experiencias latinoamericanas.

Es cierto que el grado de generalización posible para nuestros hallazgos debe ser ponderado considerando las características particulares de nuestro campo empírico. Estudiamos servicios en tres municipios con características sociodemográficas diversas: una metrópoli (São Paulo), una gran ciudad portuaria en la costa (Santos) y una gran ciudad industrial en el interior del estado (Sorocaba). Asimismo, trabajamos con UBS en territorios diversos en términos del grado de vulnerabilidad social de sus poblaciones. No obstante, es posible que en otros contextos algunos de nuestros hallazgos y discusiones no tengan el mismo sentido, considerando la diversidad regional brasileña y latinoamericana, los pequeños municipios, los contextos rurales, las poblaciones más homogéneas y/o con vulnerabilidades en salud más polarizadas (desde el punto de vista social y/o programático), así como los distintos regímenes políticos y de gobierno.

Sin embargo, considerando el interés global en un cuidado de salud integrador de las diversas dimensiones en que se experimentan los procesos de salud-enfermedad y en los recursos para la promoción, protección y recuperación de la salud, tanto colectiva como individual, entendemos que este estudio aporta insumos relevantes. En cierta medida, el contexto analizado resulta particularmente propicio para evidenciar las potencialidades y los desafíos del cuidado integral de adolescentes y jóvenes, ya que se sitúa en medio de fuertes contradicciones: por un lado, la propuesta tecnopolítica vanguardista de la integralidad, inclusiva y fuertemente apoyada en la perspectiva de los derechos humanos; por otro lado, un contexto político marcado por extremismos de derecha y el retroceso en las políticas sociales, con la ruptura del pacto social que, en la década de 1980, permitió que el Estado brasileño adoptara la propuesta del SUS y sus principios doctrinarios, como la integralidad.

En este sentido, se comprende el contraste entre la clara percepción de las y los profesionales sobre las especificidades y la complejidad de las dimensiones de las necesidades de salud adolescente, que van más allá del plano meramente morfofuncional, y la pobreza con la que estas dimensiones son incorporadas en los procesos de trabajo concretamente operados. Las necesidades que efectivamente son captadas son aquellas que se subsumen en las demandas que adolescentes y jóvenes, sus familias o instituciones llevan a los servicios de salud, y la mayoría de ellas son abordadas mediante procedimientos prescriptivos, individualizantes, fragmentados, discontinuos y centrados en aspectos fisiopatológicos. Las propuestas avanzadas de la Política Nacional de Atención Integral a la Salud de Adolescentes y Jóvenes, de 2005, que promovieron la convergencia entre las perspectivas de salud y derechos, han quedado restringidas por un retroceso que, una década después, afectó no solo a este grupo, sino a las políticas de atención primaria en general.

Esta limitación en la lectura de las necesidades se relaciona de forma dialéctica, además, con un desajuste entre los ideales de práctica de las y los profesionales y las finalidades que orientan sus acciones en el trabajo cotidiano. Si bien la promoción y prevención son consideradas por las personas entrevistadas como el horizonte del cuidado en salud de adolescentes y jóvenes, así como la necesidad de superar el enfoque individual para atender a colectivo, en la práctica, el trabajo sigue centrado en la atención a demandas clínicas y, aun en las acciones preventivas, en una dimensión biológica e individualizada. Paradójicamente, estos enfoques siguen modelos universalizantes de explicación e intervención, que resultan poco sensibles a las singularidades y aspectos contextuales de las y los adolescentes y jóvenes atendidos. Los marcadores sociales de la diferencia y sus interseccionalidades, aunque aparecen como categorías en las preocupaciones de las y los profesionales, no llegan a permear ni a diferenciar los procesos de trabajo que operan en la práctica.

Los modelos de formación de las y los profesionales, así como los modelos de atención y gestión, son identificados por las personas entrevistadas como determinantes de este desajuste, continuamente reproducido por un gerencialismo que equipara la eficacia de los servicios a su productividad en términos de un conjunto cerrado y uniformizado de actividades asistenciales. Las prácticas de prevención y promoción carecen de valorización y, por lo tanto, de profesionales, de tiempo, de espacio físico, de recursos y de reconocimiento. Esto sin mencionar el aspecto de rehabilitación y calidad de vida de adolescentes y jóvenes con discapacidad, un tema prácticamente ausente en nuestras observaciones y entrevistas.

Esta situación nos muestra cuán limitado es concebir la integralidad únicamente como una orientación técnica en la organización de la atención a la salud. Si bien tiene un componente técnico, la integralidad es también un potente intérprete de nuestro trabajo, es decir, nos permite comprender, muchas veces a través de su negación práctica en el ámbito normativo, qué estamos efectivamente produciendo (o dejando de producir) en el cuidado de adolescentes y jóvenes en la atención primaria. Cuando el horizonte normativo de la integralidad no se traduce en formas técnicas de lectura de la realidad en salud de un determinado grupo poblacional, es necesario cuestionarnos hasta qué punto ese horizonte es realmente compartido. Más aún, debemos preguntarnos si la construcción y negociación política de este horizonte incluye de hecho a sus principales destinatarios.

Esta misma potencia hermenéutica nos permite alcanzar un nivel más profundo de comprensión de las dificultades que identificamos en la implementación de la integralidad, especialmente en lo que respecta a la articulación entre diferentes servicios y sectores relacionados con el bienestar de adolescentes y jóvenes, en particular la relación entre UBS y escuela, así como las interacciones entre agentes de salud y sus destinatarios. ¿Se trata únicamente de una “falta de planificación y organización” la desarticulación entre profesionales, servicios y sectores, entre las esferas estatal y municipal, entre UBS y escuela? ¿Es accidental el hecho de que, aunque las y los profesionales de la salud sientan que tienen mucho que ofrecer, no logren convertir la UBS en un espacio de interlocución productiva con adolescentes y jóvenes? Enfrentar técnicamente estas deficiencias es una tarea ineludible para las y los profesionales de la salud y, en especial, para quienes gestionan. Sin embargo, parece fundamental ir más allá e indagar en la raíz del problema. Creemos que esto se debe al hecho de que adolescentes y jóvenes terminan convirtiéndose en diferentes “objetos” de políticas sectoriales fragmentadas, las cuales carecen de un eje unificador clave para su articulación: la presencia activa de adolescentes y jóvenes como sujetos de derecho.

Comprender cómo adolescentes y jóvenes se conciben a sí mismos como sujetos de derecho, en sus distintos contextos y experiencias cotidianas, y construir junto con ellos caminos de reconocimiento que los lleven a ocupar un espacio público de formulación e implementación de políticas para su bienestar, tal vez sean los desafíos de futuras investigaciones, a los cuales nos invita la comprensión de la integralidad, tal como ha sido reconstruida en este estudio, y el llamado que nos hace a su implementación efectiva en la práctica.
