# Supplementary material for: Atención integral a la salud de adolescentes y jóvenes desde la perspectiva de profesionales de atención primaria, San Pablo, Brasil
Source: Salud Colect. 2025 Feb 21;21:e5419. [Article in Spanish] doi: 10.18294/sc.2025.5419 (PMC12296365; doi:10.18294/sc.2025.5419)
Supplement: Translation

# Translation: Comprehensive health care for adolescents and young people from the perspective of primary care professionals, San Pablo, Brazil

**Authors:** José Ricardo de Carvalho Mesquita Ayres, Valéria Monteiro Mendes, Isabella Silva de Almeida, Macarena Urrestarazu Devincenzi, Jamile Silva Guimarães, Gabriela Junqueira Calazans, Pamela Cristina Bianchi, Alberto Shodi Yamashiro, Grupo de Pesquisa em Saúde de Jovens e Direitos Humanos (SJ&DH)9

**Affiliations:** 1 Libre-Docencia. Profesor titular, Departamento de Medicina Preventiva, Faculdade de Medicina, Universidade de São Paulo, São Paulo, Brasil. jrcayres@usp.br; 2 Doctora en Salud Pública. Posdoctoranda en Salud Colectiva, Departamento de Medicina Preventiva, Faculdade de Medicina, Universidade de São Paulo, São Paulo, Brasil. isa.almeida@alumni.usp.br; 3 Doctora en Ciencias de la Salud. Posdoctoranda en Salud Colectiva, Departamento de Medicina Preventiva, Faculdade de Medicina, Universidade de São Paulo, São Paulo, Brasil. diego.salusso@huesped.org.ar; 4 Posdoctorada en salud Colectiva. Profesora asociada, Instituto de Saúde e Sociedade, Universidade Federal de São Paulo, São Paulo, Brasil. macarena.devincenzi@unifesp.br; 5 Doctora en Salud Pública. Profesora adjunta, Faculdade de Medicina, Universidade Federal de Minas Gerais, Belo Horizonte, Brasil. mile.guimaraes@gmail.com; 6 Doctora en Medicina Preventiva. Profesora, Instituto de Psicologia, Universidade de São Paulo, São Paulo, Brasil. gajuca@usp.br; 7 Doctora en Terapia Ocupacional. Profesora adjunta, Instituto de Saúde e Sociedade, Universidade Federal de São Paulo, São Paulo, Brasil. pamela.bianchi@unifesp.br; 8 Magíster en Tecnologías y Atención a la Salud. Doctorando en Educación, Universidade Federal de São Carlos, Sorocaba, Brasil. alberto190@gmail.com; 9 Grupo interdisciplinario e interinstitucional (USP, UNIFESP, UFSCAR, UFMG, UFR/Mato Grosso), con sede en el Departamento de Psicología Social, Universidad de São Paulo, Brasil. tematico2017pst@usp.br

**Keywords:** Adolescents, Young Adult, Integrality in Health, Primary Health Care, Human Rights, Brazil

## Abstract

Comprehensiveness is a globally recommended principle for effective health care, particularly for adolescents and young people, whose needs combine biomedical aspects with sociocultural processes of subjectivity construction and citizenship exercise. However, significant challenges hinder its effective implementation. This study aims to identify the aspects of work carried out in primary health care units in the state of São Paulo, Brazil, that professionals perceive as facilitators or obstacles to providing comprehensive health care for adolescents and young people. This qualitative study, conducted between January and November 2023, involved workplace observations and semi-structured interviews with 73 health professionals. The findings highlight the predominance of decontextualized, fragmented, and prescriptive actions within a context of resource shortages and weak intersectoral coordination. The study underscores the need for adolescents and young people to participate as rights-bearing subjects in the development of intersectoral health actions and policies.

## INTRODUCTION

The integration of services and levels of care, centered on the health needs of individuals and populations within their diverse and unique health and sociocultural contexts, is globally recommended as a fundamental element in building more effective health systems. Defined as health care organized in a way that ensures individuals receive a continuum of health promotion, disease prevention, diagnosis, treatment, disease management, rehabilitation, and palliative care-coordinated across different levels and settings of care, both within and beyond the health sector, and according to their needs throughout life -there is evidence that comprehensive care improves clinical and health outcomes, enhances health literacy and self-care, increases user and professional satisfaction, boosts service efficiency, and reduces overall costs.^1^

Several Latin American countries have adopted the perspective of comprehensive care as a central axis for organizing health care. This approach encompasses various dimensions, including care management, policy formulation, promotion and protection of rights, and human resource training, particularly regarding the health of adolescents and young people. ^(^^2^^,^^3^^,^^4^^,^^5^^,^^6^

In Brazil, comprehensive care has become one of the guiding principles of the Unified Health System (SUS), in this context named “*integralidade*” (integrality). Closely linked to the principles of universality - ensuring access for all individuals living in the country - and equity - prioritizing resources based on need - the principle of comprehensive care is essential for achieving the SUS’s goals of health promotion, protection, and recovery for the Brazilian population.^7^ Although integrality is an imprecise and multifaceted principle, there is consensus that comprehensive care reflects the SUS’s commitment to a health approach that goes beyond providing assistance in cases of illness. Instead, it actively engages in health production, understood as the broad notion of well-being for individuals and communities, taking into account their diverse sociocultural contexts, legitimate values, interests, and, most importantly, their rights.^8^

If comprehensive care is essential for the health of the population as a whole, it is even more crucial for adolescents and young people, defined as those between the ages of 10 and 24. This is because the physical and psychosocial characteristics of this population segment involve complex and interrelated issues -such as bodily transformations, identity and autonomy development, sexual activity, and shifts in socialization patterns- that require a broad and sensitive approach to address their specific health policy and service needs.^9^

As the primary entry point to the SUS, primary care plays a vital role in ensuring comprehensive health care for adolescents and young people. It is both accessible and capable of resolving nearly 85% of health demands, while also being the most technologically suitable setting for delivering integrated health care.^9^ The basic health unit, through the extensive reach of the SUS, holds the greatest potential for providing appropriate health care to adolescents and young people. Within their own communities - embedded in their daily lives, sociocultural and health contexts, and in long-term interaction with other local social resources, particularly schools - there are optimal opportunities to develop response capacities that enable truly comprehensive health care for this population.

However, realizing this potential has presented challenges.^10^^,^^11^^,^^12^ After 37 years of the SUS’s existence, are health services truly providing comprehensive care for adolescents and young people in the daily work of basic health unit facilities? Are health teams sensitized, prepared, and equipped with sufficient conceptual, technical, and material resources to carry out this work?. To contribute to answering these questions, this study aims to identify the aspects of work carried out in Basic Health Units in the state of São Paulo, Brazil, that professionals perceive as facilitators or obstacles to the provision of comprehensive health care for adolescents and young people.

## METHOD

This study is part of a broader project on the vulnerabilities of adolescents and young people to STIs/HIV and intimate partner violence. The project aims to assess the feasibility, acceptability, and sustainability of health promotion and harm prevention practices from a human rights perspective. Based on the conceptual framework of vulnerability and human rights,^13^ the ongoing project has been conducted since the second half of 2019 in secondary schools in three municipalities in the state of São Paulo: São Paulo, Santos, and Sorocaba.

Building on the themes of sexual and reproductive health and violence -while progressively integrating other syndemic conditions throughout the project, such as psychosocial distress, academic difficulties, the COVID-19 pandemic, dengue, and mpox- a model called “comprehensive prevention” was developed.

In addition to proposing an integrated approach to diagnosing vulnerabilities and responding to them, the model under analysis aims to link school-based actions with the basic health unit (BHU) in the same community as the school. To this end, one of the research focal points was to assess the possibilities and limitations of these BHU facilities in working with adolescents and young people from a comprehensive care perspective. This assessment considered both the actions they were already implementing in their daily practice and the potential synergies that could be developed in coordination with the ongoing work in schools.

To achieve this, a qualitative study was conducted. While it included observations of daily work routines and semi-structured interviews with both health professionals and users of six health units across the three participating municipalities, this article focuses solely on the interviews with professionals, comparing their insights with observed aspects of service delivery.

The study included peripheral territories with varying socioeconomic patterns, ranging from areas of high vulnerability (*favelas*) to lower-middle and middle-class neighborhoods. In these contexts, access, equity, and comprehensive care are priorities due to the population’s reliance on the public health system and their strong dependence on the SUS for health promotion, protection, and recovery.

Fieldwork took place between January and November 2023. Both observations and interviews were conducted by the research team, which comprised faculty members and researchers from various universities with a multidisciplinary approach (including medicine, psychology, social sciences, occupational therapy, physical education, and nutrition). The study followed a structured guide designed to explore four analytical dimensions based on the *theory of the health work process* (THWP).^14^

Developed by Ricardo Bruno Mendes Gonçalves, one of the leading theorists behind the concepts that shaped the field of collective health,^15^ the theory of the health work process is grounded in historical materialism and engages with critical epistemology thinkers such as Canguilhem and Foucault. It challenges idealistic and ahistorical approaches that treat health practices as merely the application of scientific knowledge through techniques aimed at restoring health. Instead, the theory of the health work process seeks to examine the inherent connection between health technologies and the social and political processes of the contexts in which they are developed and applied.

By understanding health practices as work, the theory of the health work process enables the identification and analytical interrelation of the fundamental components of health technologies, characterizing their social and political significance across different local contexts and historical periods. These components can be broadly described as follows:

1. Object: The substrate on which the transformation process of work is applied.
2. Product: The intended outcome of this process, aligned with its goals.
3. Instruments: The material (artifacts, institutions) and immaterial (techniques, knowledge) resources used to achieve the work’s objectives.
4. Agents: The individuals who conceive and/or carry out work processes at different stages.

From this perspective, it is not possible to fully understand these components and their interconnections without considering the health needs that are socially and historically constructed in the contexts where these technologies operate, as well as the political factors that mediate the relationships between those who design, implement, and receive these products.

Building on the theory of the health work process, this study aims to address the concept of comprehensive care and the challenge of its operationalization in health services from *four analytical perspectives*. First, it is essential to identify the health needs that guide the work being carried out. Both health professionals and service users have some conception of what should be achieved when proposing or receiving any form of health care. Therefore, they conceptualize what *needs* to be transformed (in the body, environment, behavior, etc.).

In this sense, by understanding needs, the *goals* of the work - what is intended to be achieved as a result - are inseparably conceived. To achieve these goals, a range of material and immaterial resources must be mobilized, which help realize the work’s objectives and are organized, stabilized, and reproduced in the form of technologies (clinical, preventive, rehabilitative, and health-promoting).

With the social and technoscientific processes of the division of labor, the control and management of material and immaterial technologies are not supervised by a single agent nor limited to one workspace. Rather, they are distributed across various professional expertise and sectors responsible for their application. In this regard, health care especially when oriented toward comprehensiveness requires coordination and demands greater interdisciplinary, multiprofessional, and intersectoral *articulation*.

As a result, for all these different work operators to integrate their areas of expertise and competencies in order to serve the recipients of health actions, the *interactions* between these various actors will be of crucial importance both among professionals themselves and, especially, between professionals and the individuals receiving care.

Based on these four perspectives for analyzing health practices, the four analytical dimensions guiding the present study were defined. Table 1 summarizes, for each dimension, the guiding questions that shaped the interviews and observations within the service, as well as their interpretation.

**Table 1:** Analytical dimensions based on the comprehensiveness of work processes.

Variables	n	%
**Identidad de género**		
Feminidades trans	709	59,3
Masculinidades trans	304	25,4
Otras identidades no cis	183	15,3
**Edad agrupada**		
16-24	292	24,4
25-34	435	36,4
35-44	284	23,7
45-54	144	12,0
55 y +	41	3,4
**Región**		
Centro	468	39,1
AMBA	410	34,3
NOA	107	8,9
Patagonia	73	6,1
NEA	72	6,0
Cuyo	66	5,5
**Máximo nivel educativo**		
Primario	230	19,2
Secundario	664	55,5
Superior	302	25,3
**Cambio registral**		
Sí	837	70,0
No	359	30,0

Source: Own elaboration.

Access to services and to the health professionals and community health agents was negotiated between the academic institutions responsible for the research and the institutions responsible for health care, both at the central level of municipal management and at the local level of the health units. The number of professionals interviewed was not predetermined, as it depended on the structure and organization of work in each unit studied. The goal was to create a panel that was as representative as possible of the different professional profiles involved in the work processes carried out in each unit.

The inclusion of at least the person in charge of managing the basic health unit, a medical professional, a nursing professional, and community health agents in the interviews was prioritized due to their strategic positions in defining and operating the work processes. However, a broader range of professionals was also included, as shown in Table 2.

**Table 2:** Profile of the services, interviewed professionals, and observed aspects. Municipalities of São Paulo, Santos, and Sorocaba, São Paulo state, Brazil, 2023.

Participating service number	Health services			Interviewed professionals			Observations
	Type	Management	Number of FHSteams	Specialty	Gender	Age at the time of interview	
Service 1 (S1)	Basic Health Unit	Social Health Organization	6	Nurse in charge	Female	38	Basic Health Unit: reception, welcoming, waiting rooms, meetings (matrix support*, Violence Prevention Hub), group activities. Services and organizations in the territory: NGO “Casa do Zezinho” (institution offering refuge during the day in out-of-school periods for children and adolescents in social vulnerability) and the Institutional Shelter Service for Children and Adolescents (SAICA).
				Nurse 1	Female	29	
				Nurse 2	Female	42	
				Physician in charge	Female	49	
				Physician 1	Male	26	
				Physician 2	Male	41	
				Physical education professional	Male	30	
				Nutritionist	Female	34	
				Psychologist	Female	29	
				Physiotherapist	Female	42	
				Dentist in charge	Female	44	
				CHA 1	Male	27	
				CHA 2	Male	43	
				Manager	Male	-	
Service 2 (S2)	Basic Health Unit	Social Health Organization	7	Occupational therapist	Female	39	Basic Health Unit: general aspects of the unit (physical space, comfort, institutional climate), consultations, home visits, meetings (technical team, nursing, Violence Prevention Hub, specialized teams for children and adolescents victims/witnesses of violence, matrix support* with the Family Health Support Center, mental health with Psychosocial Care Centers, and SAICA. Services and associations in the territory: two NGOs; Children and Adolescents Center; Youth Center; Young Apprentice Program; Cultural Association (recreational and sports).
				Psychologist	Male	37	
				Speech therapist	Female	38	
				Physical education professional	Male	43	
				Physician	Male	28	
				Physician	Female	-	
				Nurse in charge	Female	-	
				Nurse 1	Female	42	
				Nurse 2	Female	-	
				CHA 1	Female	45	
				CHA 2	Female	30	
				CHA 3	Female	57	
				CHA 4	Female	49	
				CHA 5	Female	48	
				CHA 6	Female	44	
				CHA 7	Female	35	
				Manager	Female	-	
Service 3 (S3)	Basic Health Unit	Social Health Organization	7	Psychologist 1	Female	-	Basic Health Unit: reception, waiting rooms, welcoming, groups, matrix support*, home visits. Services and associations in the territory: territorial forums, NGOs, and other services such as the Child-Adolescent Psychosocial Care Center; Coexistence and Cooperative Center; Adolescent House.
				Psychologist 2	Female	44	
				Nutritionist	Female	46	
				Speech therapist	Female	34	
				Physiotherapist	Female	37	
				Physician	Female	55	
				Physician	Male	26	
				Gynecologist	Female	32	
				Pharmacist	Female	32	
				Nurse	Male	38	
				Nurse 1	Female	41	
				Nurse 2	Female	44	
				Dentist	Female	48	
				Social worker 1	Female	50	
				Social worker 2	Female	32	
				Environmental protection worker	Female	35	
				CHA 1	Female	28	
				CHA 2	Female	39	
				Manager	Female	44	
Service 4 (S4)	Traditional Basic Health Unit	Social Health Organization	3	Nurse	Female	59	Basic Health Unit: reception, waiting room, adolescent group.
				Psychologist	Female	28	
				Social worker 1	Female	45	
				Social worker 2	Female	38	
				Pediatrician	Female	40	
				Manager	Female	42	
Service 5 (S5)	Family Health Unit	Municipal	3	Psychologist	Male	54	Family Health Unit: reception, welcoming in waiting rooms, team meetings, visits to the territory, home visits. Services and associations in the territory: specialized prenatal care for high-risk pregnancies.
				Nurse	Female	41	
				Social worker	Female	54	
				CHA	Female	49	
				Manager	Female	41	
Service 6 (S6)	Traditional Basic Health Unit	Municipal	0	Nursing technician 1	Female	53	Basic Health Unit: Waiting room, reception, pharmacy, treatment room, nursing rooms, dentistry, instrument sterilization service, consulting rooms (clinical, pediatrics, gynecology), and staff cafeteria. Services and associations in the territory: Child-Adolescent Psychosocial Care Center.
				Nursing technician 2	Female	51	
				Nursing technician 3	Female	40	
				Nursing technician 4	Female	60	
				Nursing technician 5	Female	46	
				Nurse	Male	47	
				Nurse	Female	53	
				Physician	Female	29	
				Pediatrician	Female	46	
				Manager	Female	44	
				Psychologist 1 PCC	Female	26	
				Psychologist 1 PCC	Female	31	

Source: Own elaboration. FHS = Family Health Strategy; CHA = Community Health Agents; PCC= Psychosocial Care Center. *Matrix support: a case discussion and follow-up activity by professionals from different training and/or specialties.

The health services included in the study (Table 2) combine different management models currently in place in the Unified Health System in São Paulo, as well as in much of Brazil. These services may be directly managed, meaning they are administered by the municipality in which they are located, or they may be indirectly managed by social health organizations (SHO), private entities responsible for managing and operating health units through contracts with municipalities, which transfer funds based on the achievement of performance goals.

Additionally, the units differ between those termed “traditional”, which operate with conventional teams (general medicine, pediatrics, obstetrics, etc.), typically with models of care that rely more on the active demand from users, and those organized under the *Family Health Strategy*, which consist of teams that include general medicine, nursing, nursing technicians, and community health agents. These units follow a more territorialized model of care, based on active and ongoing interaction with families and institutions within their assigned areas.^16^

Both types of healthcare units may also involve broader multiprofessional teams (dentistry, psychology, physiotherapy, occupational therapy, nutrition, physical education, etc.), depending on the varying needs and characteristics of the service network in each region.^17^

Table 2 presents a summary of the profiles of the services studied, as well as the interviewees and field observations, along with the respective designations of the informants to identify the excerpts included in the body of the article.

A total of 73 semi-structured interviews were conducted in single sessions, with an average duration of 40 minutes. Additionally, service observations were carried out over a typical week, meaning that activities of interest were observed during each of the operating periods of the basic health unit, considering their various frequencies. Observations were also made in spaces such as the waiting room, hallways, and dining area, which were included in the empirical material. The interviews were recorded and transcribed by specialized professionals, and were later reviewed by the research team. In addition, recordings made by the researchers after observing the services, along with notes from the field notebooks, were transcribed and compiled.

All of this material, which formed the discursive corpus that constitutes the empirical material of the study, was subjected to a hermeneutic (comprehensive-interpretive) treatment.^18^ That is, the framework of care integration from the perspective of vulnerability and human rights, and the theory of the health work process, was used as the comprehensive reference framework that allowed for the construction and interpretation of the discourse produced in the field. This interpretation, structured around the four analytical axes outlined earlier, in turn enriched this reference framework, creating a “hermeneutic circle” that allowed for a new level of understanding of the phenomena studied.^19^

Precautions were taken to ensure the confidentiality and anonymity of the participants, as well as to avoid situations that could cause discomfort, both during the interviews and in their written recording and storage. The research protocol was submitted to and approved by the Ethics Committee of the institution leading the project, the Institute of Psychology at the University of São Paulo (CAAE 00530918.9.0000.5561). All participants were informed and signed the terms of informed consent. Both the recorded material and the transcripts, identified by codes, are stored on a drive with exclusive access for the research team, available for third-party consultation only under the principles of public interest and good scientific practice, ensuring confidentiality and anonymity.

## RESULTS AND DISCUSSION

### Needs

As previously mentioned, the needs posed for health work are gradually incorporated and reproduced in the form of technologies that become socially legitimized. In this sense, they tend to be assumed as universal and absolute, shared by both workers and their beneficiaries at any time and place. However, in lived reality, the diverse everyday experiences of different individuals create differences in how these needs are understood and valued, leading to tensions, fissures, and/or conformities that impact the work actually performed and its transformative possibilities.

In this study, without overlooking the broader social consensus, we specifically focus on what the people working in the studied basic units think about the health needs of adolescents and young people, their vulnerabilities and demands, as well as what they perceive regarding how these adolescents and young people understand these needs.

When investigating the needs of adolescents and young people in the territories of the basic health unit, the tendency of the informants was to categorize the demands directed at the services, with a significant reference to the search for contraceptive methods, rapid testing for diagnosing STIs and pregnancy after exposure and risk situations-a common pattern of utilization for these groups in health services.^9^ The spontaneous demand for care of acute clinical discomforts and psychosocial suffering was also highlighted, confirming recent studies on this subject.^20^

Issues related to alcohol and drug use emerged as an indirect demand, typically attributed to the family context and psychosocial vulnerabilities. It was also observed that the demands were predominantly made by women, reflecting a gender-related access barrier, a phenomenon also identified in other Latin American contexts.^21^

*“I actually hardly attend to the boys. They don’t consult us. So we don’t have anything directed to them... but they don’t seek us out. It’s mostly the girls.”* (S5_Nurse)

Some of the interviewees highlighted what they consider to be an early onset of sexual activity in girls, between 12 and 14 years of age. The demand for contraceptive methods was pointed out as the main reason for seeking care by cis girls, especially in the 14 to 16-year-old age group. Among the most requested methods by adolescents and young people are the copper intrauterine device (IUD, lasting up to 10 years), medroxyprogesterone acetate (available in pills and injectable solution with a duration of three months), and the subdermal contraceptive implant of etonogestrel (ICS, lasting up to three years), because these are long-acting methods less prone to user error or failure.

The ICS is a long-acting pregnancy prevention method available in the SUS since 2022, although its distribution policies vary by municipality. It has become popular among adolescents and young people, being evaluated by professionals as highly effective. According to the Ministry of Health of Brazil,^22^ respecting municipal particularities, its offering is recommended for cis women of reproductive age between 18 and 49 years, who are in situations of high vulnerability, such as homelessness, violence, abusive alcohol and drug use, and/or immunosuppression.

However, the demand sometimes exceeds this profile, and it is even mentioned that the concern about its use does not always come from the young person herself:

*“...there are some who sit there and say nothing!* [Laughter] *And then they say, ‘I’m here to get the implant.’ And the mother is sitting next to her, and it’s the mother who speaks, you know? So you realize that... somehow they’re being influenced to do it.*” (S3_Gynecologist)

Although the information about pregnancy varied between services, the professionals generally emphasized concern about this aspect, but in a way that was not well contextualized in relation to the uniqueness of the lifestyles. In one of the accounts, the mismatch that sometimes occurs in interpreting needs related to sexual and reproductive health is highlighted, which ultimately becomes an obstacle to a more integrated approach to the contexts and perspectives of adolescents and young people:

*“...there’s something I find very striking, which is when an adolescent chooses, CHOOSES, the moment to have their first sexual relationship. It’s their first sexual act and they want to protect themselves, right? ... and when they arrive at the basic health unit and hear from nurses, doctors, and nursing technicians that... they’re too young to do that. That... that lack of care from the professionals is, for me, the worst. They’re not there to judge. If the adolescent made the choice, nothing will change... his choice. If this is the moment, and they came to seek... a contraceptive method to avoid pregnancy, accept it. Accept it and that’s it.”* (S5_Social Worker)

Psychosocial demands were widely mentioned by professionals from different areas of expertise: suicidal ideation/attempts, anxiety, self-harm, fears, sadness, difficulty sleeping, nervousness, impatience, social isolation, diagnoses of borderline personality disorder, schizophrenia, and depression.

Some of the interviewees link these demands to the social determinants of health and psychosocial vulnerabilities. Factors such as relationships with peers at school (bullying) and with family members, including the lack of dialogue, neglect in caregiving, and lack of affection, as well as violence (physical and/or psychological), violence in the community related to drug trafficking in one of the studied areas, insecurity about the future, loss of family income, fragility in the social protection network, and lack of opportunities both in education and in entering the labor market, all exacerbated by the experience of the pandemic, with feelings of loss, mourning, and social restrictions, were patterns identified in this study, in line with various other investigations in different contexts.^23^^,^^24^

*“...mental suffering during the pandemic among adolescents and young people, who stayed at home with little contact with people their age, lost years of socialization, learning various skills, and experiences. This caused them suffering. The increase in demand for mental health care is a new pandemic that is a consequence of the pandemic.”* (S3_Manager)

In addition to issues related to sexual and reproductive health and mental health, demands related to self-image (dermatological problems, breast reduction due to orthopedic repercussions, body standardization), gender (affirmation and transition), divergent beliefs and moral values, and nutrition were also highlighted.

Social markers and their intersectional aspects are rarely considered in educational approaches aimed at addressing various demands:

*“Health education is the foundation for prevention.* [...] *I believe that this* [referring to neglect in self-care] *is much more related to the lack of information than to age, race, gender, orientation, or religion.”* (S3_Nurse)

Concerns about school life are a very relevant aspect for the comprehensiveness of care for adolescents and young people. In this regard, professionals highlight demands related to learning difficulties, sociability, and neuropsychomotor development, though not articulated with the work processes proposed by the unit, but rather as spontaneous demands:

*“...The school has been bringing cases, and we have tried to solve them. Some more serious, others lighter. There’s also this issue of autism, which has increased a lot; many were diagnosed with autism, others with ADHD, others with hyperactivity. And thanks to the teachers, who have also spoken out, and the supervisor, who has brought these cases to the meetings...”* (S5_Community Health Agent)

### Goals

In the development of actions directed at adolescents and young people in primary care, one of the key challenges for services relates to the transition from individual care to broader and more collective care, based on health promotion and reducing vulnerabilities to diseases. The narratives and observations revealed that there are some actions in this direction within the units, but they are not specifically targeted at this group, although some professionals emphasized the importance of initiatives in this perspective:

*“Our concern in working with children, adolescents, and young people is to transition from this individual care model to broader possibilities that involve health promotion, harm prevention, and a series of issues that are arising.”* (S5_Psychologist)

Although health promotion and disease prevention are concepts that underpin work in primary care, various factors (such as outsourcing management through contracts with social health organizations, the reduction or absence of more critical training spaces focused on ongoing education for teams, and underfunding of the SUS) have significantly contributed to the strengthen­ing of a preventive approach focused on indi­vidualized prescriptions of “lifestyle changes.”

From this perspective, it is noteworthy that the predominant goals observed in the work processes in the studied health units are related to the prescription of contraceptive methods and the performance of tests, as well as addressing specific demands, as noted in the previous section.

Group activities are among the strategies most mentioned by the interviewed professionals for welcoming and following up with adolescents and young people. However, various operational difficulties were highlighted that hinder these activities, and many of the interviewees stated that there are no specific programs for this group within the services. Some professionals identified the need to incorporate other strategies for addressing prevention and health promotion, with the perspective that a group for adolescents and young people could become a safe and attractive space for health guidance, since, as other studies have shown, adherence of adolescents and young people to group educational activities is typically low.^25^

Regarding sexual and reproductive health, the need to develop activities aimed at fostering a critical perspective on the information that adolescents and young people access on the internet was highlighted. Among the topics identified by professionals as associated with misinformation for this group is the widely circulated use of the ICS contraceptive method:

*The information circulates* […] *the friend got it, someone else got it, and they want it too* […] *Most of them already know what it is. They call it “the chip.”* (S3_Gynecologist)

The activities carried out in the school context are mostly related to campaigns, primarily vaccination campaigns, conducted by nursing professionals under the challenging framework ^26^ of the Health in School Program. This program is part of health and education policies with the aim of promoting health and comprehensive education in schools under the responsibility of basic health units.

*“There are things we act on actively.* […] *We receive requests for active vaccination search, for example, checking the vaccination card, updating the vaccination declaration. During the pandemic, we conducted serology tests for teachers and serological surveys.*” (S1_Nurse RT)

Furthermore, previous experiences in the school environment were mentioned, offering clues about the effectiveness of actions integrated with the health sector, as well as their limitations and potential paths in terms of the format and content of these actions.

*“From my experience, there are many things we can do, but I don’t have any schools in my area. In another service where we worked, we carried out deworming treatments, talks about head lice, scabies. With older children, we talked about teenage pregnancy. This group of talks also covered specific topics of interest for adolescents. They are wonderful! We also provided medical coverage during the school’s sports day...”* (S1_Doctor 2)

### Articulations

A first level of articulation for the implementation of comprehensive care refers to the team itself, to its ability to recognize and integrate different forms of knowledge and technical competencies to adequately interpret and respond to health needs. In this sense, it is important to highlight two aspects that hinder this articulation: the priorities of management, which do little to support the ethical-political formation and strengthening of teams, given the reduction or even absence of collective spaces to analyze and plan work, and the lack of priority in hiring complete teams with comprehensive work schedules, leading to professionals working reduced hours and/or splitting their time between multiple health units.^27^

These movements are closely related to the fragmentation of perspectives that still predominates in health services and to the progressive reduction of spaces for dialogue and agreements that allow teams to critically examine the actions developed or proposed and how to sustain them. From the perspective of comprehensive care, this would also require the active participation of adolescents and young people.

*“There needs to be more communication between professionals and alignment, connecting the information. Maybe create a singular project so that it makes sense in that context. If we had more time for support meetings, care for this group, or for any patient, would be more effective, right?* […] *Everyone’s schedule is overloaded. We used to have two support meetings per month for each team. Now it’s one every 15 days for all teams. The health agents used to participate as well. Now only the nurse is present. We’ve lost the richness of the details the health agents contributed. Sometimes they know the person since birth.”* (S1_Nutritionist)

Regarding the articulations between different levels of health care and between different sectors of activity, there is recognition of the existence of resources and, as in other contexts, their importance. However, in some cases, limitations are identified regarding the recognition of existing alternatives and/or difficulties in their effective implementation:

“The integration that exists and the size of the municipal network, the number of services... it’s just a matter of getting to the right place, and then it will be solved. Many times the problem is not the network, many times it’s the professional. The network is there, but the professional doesn’t know how to use it. […] It’s a lack of knowledge of the network, or a lack of empathy, or a lack of will to act.” (S1_Doctor 2)

“*In the network meeting, all the services involved with that person participate... the school, the guardianship council, if applicable, the CREAS* [Specialized Reference Center for Social Assistance], *the CRAS* [Social Assistance Reference Center]... *we call together the entire network involved in that situation*.” (S5_Manager)

Regarding the articulation between the basic health unit and the school, an intersectoral collaboration modality that has proven to be essential for comprehensive care for adolescents and young people and their main needs,^28^^,^^29^^,^^30^ the interviewees confirm that this is a powerful path to develop, emphasizing the fact that the adolescent “is already in school,” “is in their group,” which can favor more enriching encounters and readings of their needs. However, they note that knowledge of the schools in the area is generally limited, although there are actions developed in collaboration, whether due to specific demands from the school or initiatives from the basic health unit:

*“The schools are part of our facilities. Some are more open than others. There are things we actively work on... Now we’ve resumed the PSE* [Health in School Program]. *Some topics are suggested by the PSE. Our APA, the Environmental Protection Agent, has a job at the EMEI* [Municipal Early Childhood Education School].*”* (S1_Responsible Nurse)

However, the teams identify some important obstacles to this articulation:

1) High number of schools and patients in the catchment area:

“*The school, in itself, works more on the oversight of vaccination records because we can’t cover everything, there are too many schools, we have too many patients... Even though we want to do more, there’s no time*.” (S3_Community Health Agent 1)

2) Lack of coordination for joint planning between health and education at both municipal and state levels:

*“The municipality doesn’t have a direct association with state schools, it works more with municipal ones. So, our relationship is more with the principals… One difficulty is the lack of agreement between the municipality and the state. They don’t communicate, and that impacts us a lot, right? Sometimes there are actions... We have to implement the PSE* [School Health Program]*, and I direct the dentist, the dental area, to do anthropometry, activities... but I can only send to the municipal schools. Those are the only ones that are registered.”* (S4_Manager)

3) Absence of Intrasectoral Integration (Municipal and State Health):

*“There’s no coordination. The state doesn’t communicate with the municipality. If I have a patient hospitalized in the* [state hospital]*, no one sends an email to the basic health unit* [informing]*”* (S3_Doctor)

4) Work Organization Centered on Individual Care:

*“I think schools would open their doors, but the problem, as always, is the schedule, having a time slot, and having the professionals available.* [...] *Outside of patient care, we only have home visits for bedridden patients. We, the doctors, don’t have any other external activities, nothing. I still participate in a smoking cessation group, but I can hardly attend because it clashes with my schedule.”* (S1_Lead Doctor)

There was also the identification of a certain imposition regarding the participation of adolescents and young people in the basic health unit-school integration:

*“It’s different when I invite an adolescent to a group at the basic health unit, maybe they’ll come, maybe they won’t: ‘I’m too lazy to go to the basic health unit to listen to someone talk’... It’s different if I go to the school, whether they like it or not, they’ll listen to something because they’ll be kind of forced to participate. We’ll achieve something.”* (S1_Lead Doctor)

Another obstacle for basic health unit-school integration relates to the curriculum and inflexible schedules in schools, especially considering that, as emphasized, adolescents and young people, as well as the schools themselves, seek health services episodically. Moreover, when collaborations do occur, they are limited, beyond vaccination, to giving lectures.

“*…a one-hour lecture won’t change habits for life. It’s necessary to educate so that people can also spread that information.* […] *When we approach schools to form a group, the issue always came up that there was no space in the schedule. The class timetable was already set.*” (S3_Manager)

In the current context of rising conservatism, the situation in schools refers to a prohibition on debating topics considered sensitive for families of students (especially sexuality and gender), as well as the difficulty of including issues relevant to young people:

*“What falls outside the curriculum is treated as problematic, there is a tendency to avoid the subject. Any approach that goes beyond the biological, the ‘hygienic,’ generates conflicts.”* (S3_Manager)

*“The school recognizes the need to talk about sexuality, but parents don’t want it. So, they end up limiting the topic. Once, I prepared a talk about the human body because I couldn’t talk about sex. And in that context, you try, in a very subtle way, to guide them, because they are adolescents who ask tricky questions. Parents don’t see the need, but they have a thousand doubts!”* (S2_Nurse 1)

Table 3 presents a summary of the facilitating factors and challenges related to the basic health unit-school articulation. Intersectoral, interinstitutional, and broad community articulations emerge in the contemporary scenario as fundamental components for the implementation of integrated health care for adolescents and young people in various contexts across the Americas.^5^^,^^28^^,^^30^

**Table 3:** Facilitators and obstacles for health-school integration, according to the professionals interviewed. Municipalities of São Paulo, Santos, and Sorocaba; State of São Paulo, Brazil, 2023.

Facilitators	Obstacles
The implementation of health actions in schools is facilitated by the fact that it is a trusted space for adolescents.	Overloaded work schedules (most frequently cited issue).
Health actions in schools can become more effective as professionals get closer to the adolescents.	Insufficient number of health professionals for the development of actions/programs in schools.
A more collective approach to issues related to the lives of adolescents and young people is facilitated by the knowledge of the needs experienced in the territories.	Scheduling of activities in schools versus the working hours of health professionals in basic health unit (especially those in multiprofessional teams working until 1 pm or across multiple basic health unit locations).
Schools and basic health unit have open doors (access between spaces), which facilitates the construction of dialogues and joint initiatives.	Limited coordination for the development of proposals and agreements between basic health unit and schools: basic health unit work is linked to the municipal level, while schools for adolescents and young people depend on the state level.

Source: Own elaboration.

In our study, in addition to schools, other institutional resources in the territories that work with young people and can be valuable allies of the basic health unit were mentioned. Teams such as the Institutional Reception Services for Children and Adolescents (IRSCA), the Centers for Children and Adolescents (CCA), the Youth Centers (YC), and various non-governmental organizations (NGOs) develop cultural, sports, and professional training activities, which can be crucial for the coordination of health actions and the expansion of the health promotion network for adolescents and young people.

*“*[NGO X] *is great* [...] *because everything is top-notch, right?* [...] *And they have activities in gastronomy and IT, in addition to other activities. They also work a little on life projects.”* (S2_Psychologist)

*“We had a group of adolescents at the [community] radio station.* […] *It would fill up with adolescents! Because it was a radio, there was music, rap, samba, funk. They had a workshop, and we did group activities with them there, in a whole room dedicated to graffiti.* […] [The radio managers] *found a way to attract these adolescents and get them to take responsibility for each activity.”* (S3_Nurse 1)

### Interactions

The dialogue between health professionals and the recipients of their services is a necessary condition for ensuring that the goals and methods of care are in line with the unique aspects of the needs that justify them and, therefore, for ensuring that integrality can be effectively incorporated into the work processes.^31^

The interviews revealed the perception that adolescents and young people are considered a difficult group to interact with, as they rarely attend health services and show little interest in prevention and health education activities. In this context, the testimony of one professional offers important insights into the need for teams to develop other ways of interacting with adolescents and young people:

“*I think we should reflect a little more on how the basic health unit can be more welcoming for adolescents. They come here when they need to or when their parents bring them. How much have we, as a team, been able to better train ourselves to serve without imposing our own life stories, our individual beliefs? I’ve seen many situations where people are judged. In a group of pregnant women, where there were adolescents, a dentist arrived to provide guidance and asked, ‘How old are you?’ They answered, and he said, ‘Whew, it’s early to have children, right?’ How is that adolescent going to come back to this basic health unit? We need to think more about this and offer a bit more welcome to them, maybe in the way they enter the service… Today, we have a group that works in the morning. But what about adolescents who study in the other shift? We are not covering all the hours. We need to build this more together with them*.” (S2_Occupational Therapist)

Along the same lines, a nurse emphasized the need to work with the technical team and provide greater incentives to create spaces that foster other forms of interaction, dialogue, access, and welcome for adolescents and young people, as:

“*Health professionals don’t receive much training on this in university; they don’t come with this focus on how to approach without judging.*” (S1_Nurse)

When asked about aspects that could be improved in the care of adolescents and young people and the challenges faced, the professionals pointed out various elements, including the work process. They referred to the need for a broader perception of adolescents and young people seeking health services, aiming to understand how their needs and demands manifest and impact their lives, going beyond the demand-behavior model.

*“Sometimes, if an adolescent goes to the vaccination room, does the professional there have that perspective? Maybe we could take advantage of these situations that already attract this group in some way. Vaccination is one of them because they need to be up to date, and there is a connection to school, right? It would be useful to train the professionals in the vaccination room so that when an adolescent arrives, they can ask, ‘When was the last time you had a check-up?’ in order to try to bring this adolescent, somehow, into our service.”* (S2_Doctor)

This involves sensitivity and openness to dialogue in which adolescents and young people who may arrive with a request for psychology counseling, a school-related demand, a pregnancy test request, or a vaccination action are not judged. In this way, individual and collective movements can be created to question: what approaches and dialogues can be built for more comprehensive care?

*“One example of this: I attended a young person, I think around my age, who was very sad about life. So, the consultation took a little longer, right? And we ended up delaying the whole schedule* […]. *You talk to him, explain, offer measures, support, because they believe that the doctor is just here to medicate, prescribe, and refer. When we do this, we show them that we’re not just here for that. We’re here to support them, to be there for them. I recommend a follow-up to all the patients I attend. That way, they see that I care about them.”* (S3_Doctor)

In addition to the need for greater openness and other ways of approaching adolescents and young people, the importance of interaction with families was also emphasized to facilitate the identification of their demands and the construction of more integrated work with the community, especially with the support of community health agents.

*“We don’t know much about the demand of other professionals, right? It’s a bit complicated to talk about that. For example, they* [community health agents] *do a visit once a month... we could be a bit closer. I believe that if we manage to strengthen that base, with the health agents, who are crucial for us, being closer to the family, we could better capture some of the adolescents’ demand.”* (S1_Physical Education Professional)

The accounts from community health agents revealed more dynamic movements that extend beyond the walls of the basic health unit and can open pathways for building trust between adolescents and young people, facilitating their approach to the work carried out in primary care.

*“I believe that we, the health agents, are sought after much more than the health unit itself, more than the adolescent’s arrival here* […]. *There was a case of an adolescent who had an inflamed penis with secretion, and didn’t talk to his mother. He showed me a photo and had the freedom to show it to me. He came here* [to the basic health unit] *based on my guidance. He knows that I go to his house, he knows that I work at the health center. Suddenly, he felt more trust, a stronger bond.”* (S3_Community Health Agent)

*“As I’m still new, I rely a lot on the medical history to get to know the patients during the first consultation. Usually, the ones who bring this information are the health agents... I believe they are the main source of information about patients in primary care.”* (S1_Doctor 1)

Regarding aspects of the work process that impact the care of adolescents and young people, the previously mentioned focus on meeting goals based on quantitative indicators of individual care has impoverished interactions with community spaces, limiting the understanding of health needs through more dynamic interactions in the territories.

An illustrative example of this distance was found when trying to obtain more detailed information about the number of adolescents and young people registered in the basic health unit, or about the characteristics of this population in its coverage area. The most common responses were that the data was scarce or that *“it was not available at the moment”.* It was also evident in the recurrent statements from the interviewees that the data and information used to understand adolescents and young people were limited to the medical history.

This distance undoubtedly hinders the understanding of health vulnerabilities and their relationship to the promotion and protection of the rights of adolescents and young people in the studied territories, which is reflected in the almost non-existent reference to these aspects in the interviews and observed activities. In turn, this compromises the approach to the ideal of comprehensive care.

These findings align with previous studies that have shown that the way access to basic health unit is organized (appointment scheduling, waiting time for consultation, queues for emergency care) does not favor the arrival of adolescents and young people to the services or their search for health care^32^. Short consultation times, the lack of empathy from professionals, and the predominance of biomedical practices also contribute to the lack of engagement of adolescents and young people with health services.^32^^,^^33^^,^^34^^,^^35^

The observations from the professionals coincide with findings from several studies that highlight the absence of a differentiated approach towards adolescents and young people, the persistence of negative, prejudiced, and adult-centric views, as well as practices that do not promote autonomy, self-care, or a preventive attitude in adolescents and young people, weakening the guarantee of their rights.^33^^,^^35^^,^^36^

Interactions between professionals are also affected in this work model, where team activities tend to become fragmented and isolated. Although shared consultations and meetings with the multiprofessional team were valued, joint activities depend on informal arrangements based on individual initiatives and situational possibilities, such as coinciding schedules and commitments.

*“In some ways, we create it individually and pass it on very informally. It’s more of a hallway conversation, calling a colleague to talk. Or saying, ‘come in here, join my group.’* […] *We try to do it as much as possible. For example, if a patient misses an appointment or if I see a gap in the schedule, I sit down with another professional and discuss the case, or if I have a patient who needs another specialty, I look for the professional and talk to them. It’s not something very rigid: ‘If there’s no time, it’s not done.’”* (S3_Nutritionist)

## FINAL CONSIDERATIONS

Our results highlight how challenging the concrete implementation of the abstract principle of comprehensiveness in health care is, particularly regarding the care of adolescents and young people in primary care, which corroborates other Latin American experiences.

It is true that the degree of generalization of our findings should be weighed considering the particular characteristics of our empirical field. We studied services in three municipalities with diverse sociodemographic characteristics: a metropolis (São Paulo), a large port city on the coast (Santos), and a large industrial city in the interior of the state (Sorocaba). Additionally, we worked with basic health unit in territories with different levels of social vulnerability in their populations. However, it is possible that, in other contexts, some of our findings and discussions may not carry the same meaning, considering the regional diversity of Brazil and Latin America, small municipalities, rural contexts, more homogeneous populations, and/or those with more polarized health vulnerabilities (from a social and/or programmatic point of view), as well as different political and government regimes.

Nevertheless, considering the global interest in integrative health care that encompasses the diverse dimensions in which health - illness processes are experienced and in the resources for the promotion, protection, and recovery of health - both collective and individual - we believe that this study provides relevant insights. To some extent, the context analyzed is particularly suited to highlight the potential and challenges of comprehensive care for adolescents and young people, as it is situated amid strong contradictions: on one hand, the cutting-edge technopolitical proposal of comprehensiveness, inclusive and strongly supported by the human rights perspective; on the other hand, a political context marked by right-wing extremism and setbacks in social policies, with the breakdown of the social pact that, in the 1980s, allowed the Brazilian state to adopt the SUS proposal and its doctrinal principles, such as comprehensiveness.

In this sense, we understand the contrast between the clear perception of the professionals about the specificities and complexity of adolescents’ health needs, which go beyond the purely morphofunctional aspect, and the poverty with which these dimensions are incorporated into the concrete work processes. The needs that are actually captured are those that are subsumed under the demands brought to the health services by adolescents and young people, their families, or institutions, and most of them are addressed through prescriptive, individualizing, fragmented, discontinuous procedures focused on physiopathological aspects. The proposals put forward by the National Policy for Comprehensive Health Care for Adolescents and Young People in 2005, which promoted the convergence of health and rights perspectives, have been restricted by a setback that, a decade later, affected not only this group but primary care policies in general.

This limitation in the understanding of needs is dialectically related to a mismatch between the ideals of the professionals’ practice and the goals guiding their daily actions. While promotion and prevention are considered by the interviewees as the horizon of health care for adolescents and young people, as well as the need to move beyond an individual approach to a collective one, in practice, the work remains focused on addressing clinical demands, and even in preventive actions, it remains biologically and individually oriented. Paradoxically, these approaches continue universalizing models of explanation and intervention, which are not sensitive to the singularities and contextual aspects of the adolescents and young people served. The social markers of difference and their intersections, although they appear as categories in the professionals’ concerns, do not permeate or differentiate the work processes operating in practice.

The training models of professionals, as well as the care and management models, are identified by the interviewees as determinants of this mismatch, continuously reproduced by managerialism that equates the effectiveness of services with their productivity in terms of a closed and uniform set of healthcare activities. Prevention and promotion practices lack recognition and, therefore, professionals, time, physical space, resources, and recognition. This does not even take into account the aspect of rehabilitation and quality of life for adolescents and young people with disabilities, a topic practically absent in our observations and interviews.

This situation shows us how limited it is to conceive of comprehensiveness solely as a technical guideline in the organization of health care. While it has a technical component, comprehensiveness is also a powerful interpreter of our work, meaning it allows us to understand, often through its practical denial in the normative field, what we are effectively producing (or failing to produce) in the care of adolescents and young people in primary care. When the normative horizon of comprehensiveness is not translated into technical forms of reading the health reality of a specific population group, we need to question to what extent this horizon is truly shared. Furthermore, we must ask ourselves whether the political construction and negotiation of this horizon actually includes its main beneficiaries.

This same hermeneutic power allows us to reach a deeper understanding of the difficulties identified in the implementation of comprehensiveness, particularly regarding the articulation between different services and sectors related to the well-being of adolescents and young people, specifically the relationship between basic health unit and schools, as well as interactions between health agents and their beneficiaries. Is the disarticulation between professionals, services, and sectors, between state and municipal spheres, and between basic health unit and schools simply a “lack of planning and organization”? Is it accidental that, even though health professionals feel they have much to offer, they fail to make basic health unit a productive space for dialogue with adolescents and young people? Addressing these deficiencies technically is an unavoidable task for health professionals and, especially, for those managing these services. However, it seems crucial to go beyond that and inquire about the root of the problem. We believe this is because adolescents and young people end up becoming different “objects” of fragmented sectorial policies, which lack a key unifying axis for their coordination: the active presence of adolescents and young people as rights holders.

Understanding how adolescents and young people perceive themselves as subjects of rights, in their various contexts and everyday experiences, and building pathways for recognition together with them that lead them to occupy a public space for the formulation and implementation of policies for their well-being may be the challenges for future research. These challenges are brought forth by our understanding of comprehensiveness, as reconstructed in this study, and the call for its effective implementation in practice.
